# Time-Dependent Multiconfigurational Short-Range Density Functional Theory with Generalized Valence Bond Wave Functions

**DOI:** 10.1021/acs.jpca.5c04699

**Published:** 2025-09-29

**Authors:** Michał Hapka, Hans Jørgen Aa. Jensen

**Affiliations:** † Faculty of Chemistry, 49605 University of Warsaw, ul. L. Pasteura 1, 02-093 Warsaw, Poland; ‡ Department of Physics, Chemistry and Pharmacy, University of Southern Denmark, Campusvej 55, DK-5230 Odense M, Denmark

## Abstract

We present a theory and an efficient implementation of TD-GVB-srDFT, a time-dependent multiconfigurational range-separated density functional theory based on generalized valence bond perfect-pairing (GVB-PP) wave functions. In GVB-srDFT, dynamic correlation effects are incorporated via range-separation of the Coulomb potential, using tailored Kohn–Sham functionals of the density. The present implementation builds on our earlier work on TD-GVB [Hapka et al. *J. Chem. Phys.* **2022**, *156*, 174102], which employs direct Hessian techniques for both wave function optimization and linear response. We benchmark the performance of TD-GVB-srDFT for singlet and triplet excitation energies, as well as indirect spin–spin coupling constants (SSCCs). Compared to the underlying GVB-PP model, the method significantly improves excitation energies and achieves accuracy comparable to the complete active space variant, CAS-srDFT, with mean absolute deviations of 0.2 eV. The use of the generalized Tamm-Dancoff approximation (gTDA) is mandatory for reliable treatment of triplet excitations. For organic molecules, SSCCs computed with GVB-srDFT closely match those from CAS-srDFT and HF-srDFT results, whereas pure GVB-PP performs markedly worse than CASSCF for all coupling terms. Both GVB-srDFT and CAS-srDFT accurately reproduce fluorine–metal couplings in transition metal complexes, provided that gTDA is applied to singlet contributions.

## Graphical abstract

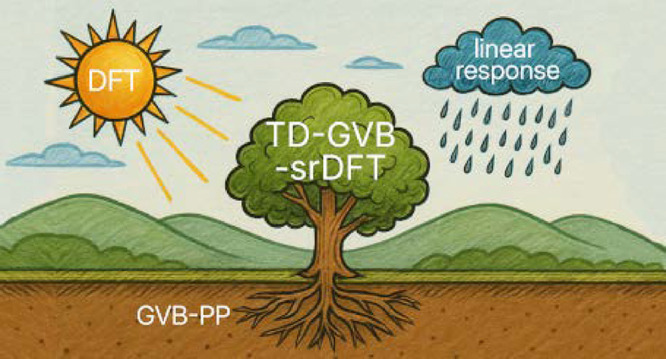

## 1 Introduction

The geminal wave function ansatz, expressed as an antisymmetrized product of two-electron functions, provides a natural extension of a single-determinant orbital description of electronic structure by explicitly accounting for electron pairing.^1,2^ Geminal models that satisfy the strong orthogonality condition, known as strictly localized geminal (SLG) wave functions,^3–5^ occupy a special place in quantum chemistry due to their relative simplicity, variational formulation, and ability to partially capture static correlation effects, giving a correct description of single bond dissociation. This work centers on one such approach: the generalized valence bond perfect pairing (GVB-PP)^6,7^ method, a special case of the more general antisymmetrized product of strongly orthogonal geminals (APSG)^6,8^ ansatz.

A fundamental limitation of SLG wave functions is their inability to describe correlation between electrons assigned to different geminals. Several strategies have been developed to recover the missing intergeminal correlation energy as a correction to the variational geminal energy. These include second-order perturbation theory,^9–15^ as well as coupled cluster (CC)-based methods, such as valence bond CC models,^16–18^ block-correlated formulations,^19,20^ a linearized multireference CC^21^ theory, and a GVB-based extension of ring CCD.^22,23^ Another class of methods^24,25^ employs extended random phase approximation (ERPA)^26^ to recover the missing part of the two-electron reduced density matrix. This approach can be derived in the framework of multireference adiabatic connection theory,^27^ and was later extended using embedding techniques to capture dispersion interactions^28,29^ and to mitigate symmetry breaking in aromatic systems.^30^

Next to wave function methods, density functional theory (DFT) has also been employed to improve the accuracy of SLG models. The straightforward approach is to supplement the SLG energy with an additional term obtained from a DFT correlation functional evaluated using the SLG density. This idea was first pursued by Kraka^31^ who combined the LDA functional^32^ with GVB-PP. Two decades later, Cagg and Rassolov^33^ proposed the APSG-DFT method, which relied on the PBE^34^ correlation energy scaled by an empirical, basis-set-dependent factor to mitigate double counting. Odoh et al.^35^ proposed an extension of GVB-PP to separated-pair approximation and combined it with multiconfigurational pair-density functional theory.^36^ Similar hybrid strategies were also explored in geminal theories with lifted strong orthogonality constraint, specifically using pair coupled cluster doubles (pCCD) wave functions.^37^ An alternative way of merging geminals with DFT was explored by Filatov et al.^38^ who developed a variant of the spin-restricted ensemble Kohn–Sham (KS) approach based on the GVB-PP ansatz.

One of the directions in the development of geminal-based methods is their extension to the time-dependent (TD) domain. The advantage of APSG linear response is the ability to access singly excited states and certain types of doubly excited states in multireference systems at low computational cost. Pioneering works in the field by Pernal and co-workers^26,39^ examined singlet excitation energies for several model molecules both in weakly and strongly correlated regimes. More recently, our study^40^ of singlet excited states on a broader set of small and medium-sized systems confirmed that, due to the absence of dynamic correlation, TD-GVB-PP provides only modest improvements over the Hartree–Fock treatment. Moreover, while the method captures certain doubly excited states, their accuracy is lower than obtained with linear-response complete active space self-consistent field (LR-CASSCF), and some double excitations are entirely absent from the spectrum. To address the missing static correlation between different electron pairs, Li and co-workers^41,42^ developed an equation-of-motion (EOM) formulation of their block-correlated CC method. When up to three-block correlations are included,^42^ the approach achieves excellent agreement with the density matrix renormalization group (DMRG) in systems that demand large active spaces.

In this work, we present an efficient implementation of a variational DFT-based approach that incorporates dynamic correlation into both the GVB-PP wave function and its linear response to time-dependent perturbations. This is achieved by combining GVB-PP with DFT via range separation of the Coulomb potential.^43,44^ In range-separated multiconfigurational DFT (MC-srDFT),^45,46^ the DFT functional is responsible for efficient description of short-range electron correlation, while the long-range wave function captures static correlation without requiring spin-symmetry breaking. Splitting the Coulomb operator not only avoids double counting of correlation in a rigorous manner, but also removes the electron–electron cusp in the long-range wave function. The immediate benefits are shorter wave function expansion and faster convergence with the basis set size.^44,47,48^

In geminal theory, range-separated APSG model and its time-dependent extension were first derived and implemented by Pernal et al.^39^ On the example of water and formaldehyde molecules, the authors demonstrated that TD-APSG-srDFT significantly reduces the errors with respect to the pure TD-APSG results in the vicinity of the equilibrium. However, TD-APSG-srDFT fails to describe dissociation curves. In the dissociation limit, the accuracy of the method is inferior to pure TD-APSG due to inadequate description of the ground state by GVB-srDFT originating from self-interaction and static correlation errors in the short-range functional.^49,50^ Garza et al.^51^ applied range-separation together with the pCCD ansatz. By incorporating the on-top pair density into short-range functionals via auxiliary spin densities,^52,53^ pCCD-srDFT predicts accurate potential energy curves for single-bond breaking, including symmetric dissociation of water.

Our aim is to provide a systematic assessment of the time-dependent GVB-srDFT model. To enable excitation energy and magnetic response property calculations, we formulate TD-GVB-srDFT for perturbations of both singlet and triplet symmetry. We compare the performance of the method against both the uncorrected GVB-PP linear response and time-dependent complete active space short-range DFT (TD-CAS-srDFT) approach. Singlet and triplet excitation energies are computed for a representative set of small- and medium-sized molecules. In addition, we examine the ability of GVB-srDFT to describe indirect spin–spin coupling constants. Beyond benchmarking on standard data sets, we present a case study of the challenging VF_6_^–^ complex.^54^ Special attention is given to the accuracy of GVB-PP relative to CASSCF for triplet-state response, which to the best of our knowledge has not been previously analyzed in the literature.

In Section 2, we present the details of the implementation, focusing on aspects specific to GVB-PP. The technical details of our calculations are described in Section 3, followed by a discussion of the results in Section 4. We conclude in Section 5 with a summary of the key results and observations.

## 2 Theory

### 2.1 GVB-srDFT Wave Function Optimization

The central concept in the MC-srDFT theory is the separation of the Coulomb operator into long-range (lr) and short-range (sr) contributions

$$\frac{1}{r}=v_{\mathrm{ee}}^{\mathrm{lr}}(r)+v_{\mathrm{ee}}^{\mathrm{sr}}(r)$$ 1

satisfying asymptotic conditions

$$\lim_{r\to 0}\;rv_{\mathrm{ee}}^{\mathrm{sr}}(r)=1,\lim_{r\to \infty }\;rv_{\mathrm{ee}}^{\mathrm{lr}}(r)=1$$ 2

The separation enables the use of a density functional to describe the short-range electron interactions, while the wave function is employed to handle the long-ranged electron interactions. The ground state energy is obtained by minimizing the range-separated density functional and is expressed as the sum of wave function and functional contributions

$$E(\lambda )=\langle \Psi^{\mathrm{lr}}(\lambda )|{Ĥ}^{\mathrm{lr}}|\Psi^{\mathrm{lr}}(\lambda )\rangle +E_{H}^{\mathrm{sr}}[\rho_{C}]+E_{\mathrm{xc}}^{\mathrm{sr}}[\xi ]$$ 3

where the long-range Hamiltonian is the sum of kinetic and nuclei-electron operators, *T̂* and *V̂*_ne_, respectively, the nuclear repulsion term, *V*_nn_, and the long-range electron interaction part

$${Ĥ}^{\mathrm{lr}}=\mathrm{T̂}+{\mathrm{V̂}}_{\mathrm{ne}}+V_{\mathrm{nn}}+{\mathrm{V̂}}_{\mathrm{ee}}^{\mathrm{lr}}$$ 4

where *V̂* ^lr^ = ∑_*j*>*i*_ *v* ^lr^(*r*_*ij*_). We follow the typical choice of representing the long-range interaction via an error function

$$v^{\mathrm{lr}}(r)=\frac{\mathrm{erf}(\mu r)}{r}$$ 5

with μ denoting the range-separation parameter. The *E*_H_^sr^ and *E*_xc_^sr^ terms in eq 3 are the short-range Hartree and exchange-correlation (xc) functionals, respectively. For generalized gradient approximation (GGA) models considered here, the latter depends on charge and spin densities, denoted ρ_*C*_(**r**, **λ**) and ρ_*S*_(**r**, **λ**), respectively, and their gradients, γ_*XY*_(**r**, **λ**) = ∇ρ_*X*_(**r**, **λ**)∇ρ_*Y*_(**r**, **λ**), where *X*, *Y* = *C*, *S*; the dependence **ξ** = {ρ_*C*_, ρ_*S*_, γ_*CC*_, γ_*SS*_, γ_*CS*_}. The **λ** vector collects the variational wave function parameters which we discuss below.

In this work, we consider long-range wave functions built from singlet coupled geminals

$$\psi_{P}({\overset{-}{x}}_{1},{\overset{-}{x}}_{2})=\frac{1}{\sqrt{2}}\sum_{p\in P}c_{p}\varphi_{p}(r_{2})\varphi_{p}(r_{1})[\alpha (1)\beta (2)-\beta (1)\alpha (2)]$$ 6

where **x̅** = (**r**, σ) is a combined spatial and spin coordinate, {φ_*p*_} and {*c*_*p*_} are natural orbitals and expansion coefficients, respectively, and *P* is the geminal index. For each geminal the coefficients are normalized ∀_*P*_ ∑_*p*∈*P*_ *c*_*p*_^2^ = 1, and relate to natural spin–orbital occupation numbers, *n*_*p*_ = *c*_*p*_^2^, where *n*_*p*_ ∈ [0, 1]. The geminals are strongly orthogonal which means that for each pair it occurs that

$$\forall_{P\neq Q}\forall_{{\overset{-}{x}}_{1},{\overset{-}{x}}_{1}^{\prime }}\int \psi_{P}({\overset{-}{x}}_{1},{\overset{-}{x}}_{2})\psi_{Q}({\overset{-}{x}}_{1}^{\prime },{\overset{-}{x}}_{2})d{\overset{-}{x}}_{2}=0$$ 7

We focus on the special case of GVB-PP wave functions where each geminal is represented by a pair of orbitals

$$\forall_{P}\psi_{P}({\overset{-}{x}}_{1},{\overset{-}{x}}_{2})=\frac{1}{\sqrt{2}}[c_{1}\varphi_{1}(r_{1})\varphi_{1}(r_{2})+c_{2}\varphi_{2}(r_{1})\varphi_{2}(r_{2})]\times [\alpha (1)\beta (2)-\beta (1)\alpha (2)]$$ 8

and the full wave function is an antisymmetrized product of *N*_G_ = *N*/2 geminals, where *N* is the even number of electrons. Note, though, that our implementation can also treat APSG wave functions, if desired.

The variational parameters of a GVB-PP wave function are geminal expansion coefficients and orbital-rotation variables. In ref 40 we introduced a geminal-norm conserving parametrization of a GVB-PP wave function

$$\forall_{p\in P}c_{p}=\frac{c_{p}^{0}+x_{p}}{\sqrt{\left|1+2\underset{q\in P}{\sum }c_{q}^{0}x_{q}+\underset{q\in P}{\sum }x_{q}^{2}\right|}}$$ 9

where the parameters *x*_*p*_ are unconstrained and ∀_*P*_: ∑_*p*∈*P*_(*c*_*p*_^0^)^2^ = 1. To describe the orbital rotations we use the antisymmetric real singlet orbital-rotation operator

$$\mathrm{\kappa ̂}=\sum_{pq}\kappa_{pq}{Ê}_{pq}=\sum_{p>q}\kappa_{pq}({Ê}_{pq}-{Ê}_{qp})$$ 10

where **κ** is an antisymmetric matrix and *Ê*_*pq*_ is the singlet excitation operator, *Ê*_*pq*_ = *â*_*p*α_^†^ *â*_*q*α_ + *â*_*p*β_^†^ *â*_*q*β_.

The GVB-srDFT wave function optimization is carried out using the restricted-step second-order optimization algorithm,^55–57^ as implemented in Dalton.^58^ The algorithm is based on second-order Taylor expansion of the electronic energy in the λ parameters, around **λ** = 0

$$E\left(\lambda \right)=E_{0}+g^{T}\lambda +\frac{1}{2}\lambda^{T}H\lambda$$ 11

where **g** and **H** are the electronic gradient and electronic Hessian, respectively, and **λ** = (***x***, **κ**) gathers the geminal coefficients and orbital rotation variables. Differentiating the GVB-srDFT energy with respect to coefficients and orbital parameters leads to a block structure of both the gradient and Hessian

$$g_{i}=g_{i}^{\mathrm{lr}}+g_{H,i}^{\mathrm{sr}}+g_{\mathrm{xc},i}^{\mathrm{sr}}=\frac{\partial E^{\mathrm{lr}}}{\partial \lambda_{i}}+\frac{\partial E_{H}^{\mathrm{sr}}[\rho ]}{\partial \lambda_{i}}+\frac{\partial E_{\mathrm{xc}}^{\mathrm{sr}}[\xi ]}{\partial \lambda_{i}}$$ 12

The Hessian is obtained as **H** = **P** **K** **P**, where **K** is the augmented Hessian for the real-valued GVB-srDFT wave function, and **P** is the projector operator that removes redundant variables in the coefficient space

$$P=1-O=1-\underset{Q}{\sum }p^{Q}{\left(p^{Q}\right)}^{T}$$ 13

where for each geminal *Q* we have introduced the redundancy vector **p** ^*Q*^

$$\forall_{Q}p^{Q}=\left(\begin{array}{l} x^{Q}\\ 0 \end{array}\right)$$ 14

$$\forall_{Q}x^{Q}=\left[c_{1}\delta_{I_{1}Q},c_{2}\delta_{I_{2}Q},\cdots \right]$$ 15

where *I*_*r*_ denotes the geminal that orbital *r* belongs to. Similar to the gradient, the Hessian contains both wave function and srDFT components

$$K_{ij}=K_{ij}^{\mathrm{lr}}+K_{H,ij}^{\mathrm{sr}}+K_{\mathrm{xc},ij}^{\mathrm{sr}}=\frac{\partial^{2}E^{\mathrm{lr}}}{\partial \lambda_{i}\partial \lambda_{j}}+\frac{\partial^{2}E_{H}^{\mathrm{sr}}\left[\rho_{C}\right]}{\partial \lambda_{i}\partial \lambda_{j}}+\frac{\partial^{2}E_{\mathrm{xc}}^{\mathrm{sr}}\left[\xi \right]}{\partial \lambda_{i}\partial \lambda_{j}}$$ 16

The algorithm avoids explicit construction of the full Hessian matrix by directly computing contributions to the Hessian using linear transformations with trial vectors **b**_*n*_

$$\sigma_{n}=PKPb_{n}=PKb_{n}=P\left(\begin{array}{ll} K^{gg} & K^{go}\\ K^{og} & K^{oo} \end{array}\right)\left(\begin{array}{l} b_{n}^{g}\\ b_{n}^{o} \end{array}\right)$$ 17

The trial vectors are chosen to obey **P** **b**_*n*_ = **b**_*n*_, to facilitate handling of the redundancies.

The GVB-PP long-range contributions to the gradient and **σ**_*n*_ elements are identical to the regular, i.e., full-range, terms described in ref 40, except that the full-range two-electron integrals are replaced with the long-range ones. The short-range exchange-correlation contributions to the gradient and Hessian are obtained analogously to the general MC-srDFT framework.^59^ Below we summarize the key points relevant to our GVB-srDFT implementation.

The orbital contribution to the sr exchange-correlation gradient depends only on the reduced one-electron charge density matrix [c.f., eqs 23–25 in ref 59] which for GVB-srDFT wave function in the NO representation takes the diagonal form

$$D_{pq}^{C}=\left\langle \Psi^{\mathrm{GVB}}\right|{Ê}_{pq}\left|\Psi^{\mathrm{GVB}}\right\rangle =2n_{p}\delta_{pq}=2c_{p}^{2}\delta_{pq}$$ 18

The geminal contribution to the gradient is the same as in the GVB-PP case,^40^ except it is evaluated using long-range two-electron integrals and a one-electron Hamiltonian modified by the addition of the short-range xc potential, which reads

$$V_{\mathrm{srxc},pq}^{C}=\int \left[\frac{\partial e_{\mathrm{xc}}^{\mathrm{sr}}\left(\xi \right)}{\partial \rho_{C}}\Omega_{pq}\left(r\right)+\left(2\frac{\partial e_{\mathrm{xc}}^{\mathrm{sr}}\left(\xi \right)}{\partial \gamma_{CC}}\nabla \rho_{C}+\frac{\partial e_{\mathrm{xc}}^{\mathrm{sr}}\left(\xi \right)}{\partial \gamma_{CS}}\nabla \rho_{S}\right)\cdot \nabla \Omega_{pq}\right]dr$$ 19

where *e*_xc_^sr^(**ξ**) is the short-range xc energy density, and Ω_*pq*_(**r**) = φ_p_^*^(**r**)­φ_*q*_(**r**). Contributions to the gradient from the Hartree term *E*_H_^sr^[ρ_*C*_] are analogous, with the short-range Hartree potential in place of the short-range xc potential.

Evaluation of the srDFT components of the Hessian, $K_{\mathrm{xc},ij}^{\mathrm{sr}}=\int \frac{\partial^{2}e_{\mathrm{xc}}^{\mathrm{sr}}(\xi )}{\partial \lambda_{j}\partial \lambda_{i}}dr$ , involves two different types of terms

$$\frac{\partial^{2}e_{\mathrm{xc}}^{\mathrm{sr}}\left(\xi \right)}{\partial \lambda_{i}\partial \lambda_{j}}=\underset{l}{\sum }\underset{k}{\sum }\frac{\partial^{2}e_{\mathrm{xc}}^{\mathrm{sr}}\left(\xi \right)}{\partial \xi_{l}\partial \xi_{k}}\frac{\partial \xi_{l}}{\partial \lambda_{j}}\frac{\partial \xi_{k}}{\partial \lambda_{i}}+\underset{k}{\sum }\frac{\partial e_{\mathrm{xc}}^{\mathrm{sr}}\left(\xi \right)}{\partial \xi_{k}}\frac{\partial^{2}\xi_{k}}{\partial \lambda_{j}\partial \lambda_{i}}$$ 20

which follows from the nonlinearity of the srDFT functional. The direct Hessian technique, eq 17, requires contracting eq 20 with the geminal and orbital trial vectors

$$\sum_{j}\frac{\partial^{2}e_{\mathrm{xc}}^{\mathrm{sr}}}{\partial \lambda_{i}\partial \lambda_{j}}b_{j}=\sum_{l}\sum_{k}\frac{\partial^{2}e_{\mathrm{xc}}^{\mathrm{sr}}}{\partial \xi_{l}\partial \xi_{k}}\frac{\partial \xi_{k}}{\partial \lambda_{i}}\left[\sum_{j}\frac{\partial \xi_{l}}{\partial \lambda_{j}}b_{j}\right]+\sum_{k}\frac{\partial e_{\mathrm{xc}}^{\mathrm{sr}}}{\partial \xi_{k}}\left[\sum_{j}\frac{\partial^{2}\xi_{k}}{\partial \lambda_{j}\partial \lambda_{i}}b_{j}\right]$$ 21

In the implementation, we restrict ourselves to closed-shell singlet GVB-srDFT reference wave functions, cf. eq 6, so that derivatives with respect to the spin density variables are not considered. Equation 21 can be cast in a convenient form using transition density matrices, $D_{rs}^{C,(1\lambda )}=\sum_{i}\frac{D_{rs}^{C}}{\partial \lambda_{i}}b_{i}$ , of two types: the one-index transformed^57,60^ density matrix for orbital trial vectors, which depends solely on the density matrix

$$D_{rs}^{C,(1o)}=\sum_{t>u}\frac{\partial D_{rs}^{C}}{\partial \kappa_{tu}}b_{tu}^{o}=\sum_{t}(D_{ts}^{C}b_{rt}^{o}+D_{rt}^{C}b_{st}^{o})$$ 22

and the transition density matrix for geminal trial vectors, which for the specific case of a GVB wave function takes the form

$$D_{rs}^{C,(1g)}=\sum_{j}\frac{\partial D_{rs}^{C}}{\partial c_{j}}b_{j}^{g}=\langle \Psi^{\mathrm{GVB}}|{Ê}_{rs}|B^{\mathrm{GVB}}\rangle +\langle B^{\mathrm{GVB}}|{Ê}_{rs}|\Psi^{\mathrm{GVB}}\rangle =4c_{r}b_{r}^{g}\delta_{rs}$$ 23

where we have introduced state vectors for the current geminal trial vector

$$\left|B^{\mathrm{GVB}}\right\rangle =\underset{j}{\sum }\left(\frac{\partial }{\partial c_{j}}\left|\Psi^{\mathrm{GVB}}\right\rangle b_{j}^{g}\right)$$ 24

These quantities are used to introduce effective potential operators which take the form

$$V_{\mathrm{srxc},pq}^{C,\left[1\lambda \right]}=\int \underset{rs}{\sum }K_{pq,rs}^{C,\mathrm{sr}}D_{rs}^{C,\left(1\lambda \right)}dr$$ 25

where $K_{pq,rs}^{C,\mathrm{sr}}$ are matrix elements of the charge component of the short-range exchange-correlation kernel [see, e.g., ref 61 for standard GGA form and eq 40 in ref 59 for srDFT]. With these effective potentials, one may define all necessary Hessian σ-vectors for wave function optimization, cf. Equation 42 in ref 59. The geminal part of σ-vectors before projection takes a simple form

σsrxc,ig′=∑j∂2Excsr∂cj∂cibjg=∑pqVsrxc,pqC,[1g]∂DpqC∂ci+∑pq,jVsrxc,pqC∂2DpqC∂ci∂cjbjg=4ciVsrxc,iiC,[1g]+4bigVsrxc,iiC 26

σsrxc,io′=∑rs∂2Excsr∂κrs∂cibrso=∑pqVsrxc,pqC,[1o]∂DpqC∂ci+∑pq,rsVsrxc,pqC∂2DpqC∂ci∂κrsbrso=4ci(Vsrxc,iiC,[1o]+Ṽsrxc,iiC) 27

where *Ṽ*_srxc_^*C*^ is the one-index transformed^57,60^ form of the gradient operator in eq 19, given as

$${Ṽ}_{\mathrm{srxc},pq}^{C}=\sum_{t}(V_{\mathrm{srxc},tq}^{C}b_{pt}^{o}+V_{\mathrm{srxc},pt}^{C}b_{qt}^{o})$$ 28

The wave function parametrization, eq 9, requires that the σ^*′*^-vectors defined in eqs 26 and 27 have to be projected onto the *x*-representation using the **P** projector introduced in eq 13: **σ** = **P σ** ^*′*^. Expressions for the orbital part of the Hessian σ-vectors are identical as in the MC-srDFT case [cf. eqs 42b and 42d in ref 59], except for the evaluation of density matrices and transition density matrices, cf. eqs 18 and 23, respectively.

Although our primary focus is on the linear response models, it is worth noticing that for the standard value of μ = 0.4 the GVB-srDFT optimization yields geminals with a more diffuse character compared to GVB-PP. In particular, the π orbitals of aromatic systems may become delocalized, as illustrated for the paradigmatic benzene molecule in Figures S1–S5 of the Supporting Information.

### 2.2 Linear Response for GVB-srDFT

The GVB-srDFT linear response equations can be written in a general matrix form^62,63^

$$\left(E^{\left[2\right],X}-\omega S^{\left[2\right],X}\right)\Lambda^{X}=-iV^{\left[1\right],X}$$ 29

where X = *C* or X = *S* for singlet and triplet properties, respectively, and **V** ^[1],*X*^ denotes external perturbation vector. Excitation energies correspond to poles of the response functions, i.e., they follow by setting the external perturbation to zero. The nonperturbed problem can be cast in a matrix form

$$\left(E^{\left[2\right],X}-\omega_{i}S^{\left[2\right],X}\right)\Lambda_{i}^{X}=0$$ 30

where Λ_*i*_^*X*^ denotes the eigenvector, and the ω_*i*_ eigenvalues are approximations to excitation energies.

In MC-srDFT, the Hessian separates as a sum of long-range and short-range contributions, *E* ^[2]^ = *E* ^[2],lr^ + *E* ^[2],sr^. The long-range contributions are identical to those given in ref 40, with the lr two-electron integrals instead of the full-range ones. Expressions for the srDFT contributions to the Hessian follow from eqs 17 and 21. In practice, linear response equations are solved using direct iterative algorithms described in ref 63.

To handle both real and imaginary orbital rotations for the solutions of response equations, a more general parametrization of orbital rotation operators is required^64^

$${\mathrm{\kappa ̂}}^{C}=\sum_{p\neq q}\kappa_{pq}{Ê}_{pq}$$ 31

$${\mathrm{\kappa ̂}}^{S}=\sum_{p\neq q}{\overset{-}{\kappa }}_{pq}{\mathrm{T̂}}_{pq}$$ 32

where *T̂*_*pq*_ = *â*_*p*α_^†^ *â*_*q*α_ – *â*_*p*β_^†^ *â*_*q*β_ is the triplet one-electron operator. With these general operators, the one-index transformed matrices take the form

$${Ṽ}_{\mathrm{srxc},pq}^{X}=\sum_{t}(V_{\mathrm{srxc},tq}^{X}b_{pt}^{o}-V_{\mathrm{srxc},pt}^{X}b_{tq}^{o})$$ 33

for both X = *C* and X = *S*. Note that eqs 10 and 28 are recovered for κ_*pq*_ = −κ_*qp*_ and *b*_*pq*_^*o*^ = −*b*_*qp*_^*o*^, i.e., when restricting κ̂ to real orbital rotations.

Since triplet response equations are solved here assuming a real singlet reference wave function, all quantities which are of an odd order dependency on the spin density variable *S* do not contribute to the srDFT part of the Hessian, as defined in eq 21 [see also eq 30 in ref 65]:

$$\begin{array}{ccc} \rho_{S}=0, & \gamma_{SS}=0, & \gamma_{CS}=0,\\ \nabla \rho_{S}=0, & \frac{\partial e_{\mathrm{xc}}^{\mathrm{sr}}}{\partial \rho_{S}}=0, & \frac{\partial e_{\mathrm{xc}}^{\mathrm{sr}}}{\partial \gamma_{CS}}=0,\\ \frac{\partial^{2}e_{\mathrm{xc}}^{\mathrm{sr}}}{\partial \rho_{C}\partial \rho_{S}}=0, & \frac{\partial^{2}e_{\mathrm{xc}}^{\mathrm{sr}}}{\partial \gamma_{CC}\partial \gamma_{CS}}=0, & \frac{\partial^{2}e_{\mathrm{xc}}^{\mathrm{sr}}}{\partial \gamma_{SS}\partial \gamma_{CS}}=0,\\ \frac{\partial^{2}e_{\mathrm{xc}}^{\mathrm{sr}}}{\partial \rho_{C}\partial \gamma_{CS}}=0, & \frac{\partial^{2}e_{\mathrm{xc}}^{\mathrm{sr}}}{\partial \rho_{S}\partial \gamma_{SS}}=0, & \frac{\partial^{2}e_{\mathrm{xc}}^{\mathrm{sr}}}{\partial \rho_{S}\partial \gamma_{CC}}=0 \end{array}$$ 34

The srDFT components of the Hessian for triplet response are conveniently expressed via effective triplet potential operators

$$V_{\mathrm{srxc},pq}^{S,\left[1\lambda \right]}=\int \underset{rs}{\sum }K_{pq,rs}^{S,\mathrm{srxc}}D_{rs}^{S,\left(1\lambda \right)}dr$$ 35

where $K_{pq,rs}^{S,\mathrm{srxc}}$ are matrix elements of the spin-component of the short-range xc kernel,^54^ and the spin transition density matrices $D_{rs}^{S,\left(1\lambda \right)}=\underset{i}{\sum }\frac{D_{rs}^{S}}{\partial \lambda_{i}}b_{i}$ are defined with respect to the spin-density matrix *D*_*pq*_^*S*^ = ⟨Ψ^GVB^|*T̂*_*pq*_|Ψ^GVB^⟩. Note that for a singlet-reference wave function **D** ^*S*^ = 0, but **D** ^*S*,(1λ)^≠0. Since our implementation of the GVB-PP wave function does not include triplet components, i.e., geminals in eq 6 are singlet coupled, the geminal part of the effective triplet potential is equal to zero, **V**_srxc_^*S*, [1*g*]^ = 0. In consequence, the only nonvanishing contributions to the Hessian σ-vectors come from the orbital trial vectors. These read

$$\sigma_{pq}^{S,o}=\sum_{r\neq s}\frac{\partial^{2}E^{\mathrm{lr}}}{\partial {\overset{-}{\kappa }}_{rs}\partial {\overset{-}{\kappa }}_{pq}}b_{rs}^{o}=\langle \Psi^{\mathrm{GVB}}|[{\mathrm{T̂}}_{pq},{\mathrm{H̃̂}}^{\mathrm{lr}}]|\Psi^{\mathrm{GVB}}\rangle$$ 36

$$\sigma_{\mathrm{srxc},pq}^{S,o}=\sum_{r\neq s}\frac{\partial^{2}E_{\mathrm{xc}}^{\mathrm{sr}}}{\partial {\overset{-}{\kappa }}_{rs}{\overset{-}{\kappa }}_{pq}}b_{rs}^{o}=\langle \Psi^{\mathrm{GVB}}|[{\mathrm{T̂}}_{pq},{\mathrm{V̂}}_{\mathrm{srxc}}^{S,[1o]}]|\Psi^{\mathrm{GVB}}\rangle$$ 37

where *H̃̂* ^lr^ is the one-index transformed lr Hamiltonian, and *V̂*_srxc_^*S*, [1*o*]^ = ∑_*pq*_ *V̂*_srxc, *pq*_^*S*, [1*o*]^ *T̂*_*pq*_. Note that eq 37 does not contain contribution from the one-index transformed sr-xc gradient, since $\frac{\partial e_{\mathrm{xc}}^{\mathrm{sr}}}{\partial \rho_{S}}=0$ . Equations 36 and 37 are computed as in the MCSCF case, see, e.g., eq 53 in ref 55. For completeness, in the Supporting Information we also provide explicit expressions for contributions to the σ-vectors in the singlet linear response equations.

### 2.3 Indirect Nuclear Spin–Spin Coupling Constants

The nonrelativistic indirect spin–spin coupling tensor describes the correction to the interaction between naked nuclear dipoles due to the presence of the electron cloud. It can be represented as a sum of four contributions^66,67^

$$J_{\mathrm{KL}}=J_{\mathrm{KL}}^{\mathrm{DSO}}+J_{\mathrm{KL}}^{\mathrm{PSO}}+J_{\mathrm{KL}}^{\mathrm{SD}}+J_{\mathrm{KL}}^{\mathrm{FC}}$$ 38

where the diamagnetic spin–orbit (DSO) and paramagnetic spin–orbit (PSO) terms arise from the coupling of nuclear magnetic moments to the orbital motion of the electrons, whereas the spin-dipole (SD) and Fermi-contact (FC) terms result from the coupling of the nuclear magnetic moments to the spin of the electron. The DSO term is evaluated as an expectation value. The remaining contributions are recovered in second-order perturbation theory from linear response functions at zero frequency: the PSO term requires solving singlet response equations; both FC and SD hyperfine tensors follow from triplet linear response functions. For the specific form of the corresponding property gradient vectors, see, e.g., ref 68.

From the isotropic part of the coupling tensor, $J_{\mathrm{KL}}=\frac{1}{3}\mathrm{Tr}(J_{\mathrm{KL}})$ , one recovers the indirect spin–spin coupling constant, which we focus on in this work. We have integrated our GVB-srDFT linear response codes with the SSCC implementation available in Dalton, originally developed for MCSCF wave functions.^68^

## 3 Computational Details

The second-order GVB-srDFT wave function optimization and TD-GVB-sr-DFT linear response equations were implemented in a development version of the Dalton program.^58,69^ For all systems, initial geminals were generated using our black-box, two-step scheme, described in ref 40. The starting strongly occupied orbitals were obtained by localizing relevant SCF orbitals via the Foster-Boys^70^ scheme. GVB-srDFT calculations were performed with short-range LDA (srLDA)^71^ and short-range PBE (srPBE)^72^ exchange-correlation functionals. The computational cost of GVB-srDFT wave function optimization is identical to GVB-PP scaling for the long-range GVB-PP part, plus DFT scaling for the srDFT part, as the total energy is the sum of two contributions. Analogously, the scaling of TD-GVB-srDFT response equations corresponds to the combined scaling of the TD-GVB-PP and TD-DFT parts.

The data set of reference excitation energies is identical to that used in ref 65, comprising 33 molecules with geometries taken either from the Thiel’s set^73,74^ (16 molecules) or from the set of Loos et al.^75^ (17 molecules). The data set includes 110 singlet and 110 triplet excitation entries, with reference values taken from CC3 calculations as reported in refs 74 and 75. The LDA, PBE, CASSCF and CAS-srDFT data for triplet excitations were taken from ref 65 (for the active space selection, see the Supporting Information of ref 65). For singlet excitations, we employed the TZVP basis set^76^ for systems of the Thiel’s data set and the aug-cc-pVTZ basis set^77^ for the remaining molecules. For all triplet excitations, the aug-cc-pVTZ basis set was selected. In our previous work, we established that GVB-PP fails to recover certain singlet doubly excited states from the Thiel set—specifically, the 2^1^ *A*_g_ states of butadiene, hexatriene and octatetraene, as well as the 2^1^ *A*_1_ state of cyclopentadiene.^40^ Since these will also be absent at the GVB-srDFT level, we exclude them from the benchmark set across all methods to ensure a consistent comparison.

All GVB-srDFT calculations were performed employing the full GVB-PP valence geminal space. In the Supporting Information, we also provide excitation energies obtained with a smaller active space selected based on MP2-srDFT natural occupation numbers, analogously to the CAS-srDFT active space defined in ref 65 [see Table S1 for the GVB-srDFT­(μ = 0.4) active space composition].

The data set of reference SSCCs by Faber et al.^78^ analyzed in Section 4.2 comprises 45 data points for 13 small single-reference molecules computed at the CC3/aug-ccJ-pVTZ level of theory. The KS-DFT, CASSCF and CAS-srDFT data for SSCCs were taken from ref 54. All GVB-PP and GVB-srDFT calculations of SSCCs were performed in the aug-ccJ-pVTZ basis set.^79^

## 4 Results

### 4.1 Singlet and Triplet Excitations

Table 1 and Figure 1 present error statistics for singlet excitations. As reported in ref 40, GVB-PP systematically underperforms compared to CASSCF, with both mean absolute deviations (MADs) and mean signed deviations (MSDs) larger by about 0.2 eV. When dynamic correlation is included via a short-range DFT functional, the MAD decreases from 0.7 eV for GVB-PP to 0.2 eV for GVB-srDFT, matching the accuracy of CAS-srDFT. The srLDA and srPBE models perform essentially the same when combined with either GVB-PP or HF; for CASSCF, srPBE offers slightly better accuracy. Since molecules in the set do not feature strong correlation effects, the results for HF-, GVB- and CAS-srDFT are generally similar. Still, both GVB-srDFT and CAS-srDFT reduce the error spread relative to HF-srDFT, with the standard deviation dropping from 0.3 to 0.2 eV. On average, GVB-srDFT exhibits identical mean errors as CAS-srDFT. However, for states with a more pronounced doubly excited character, as indicated by the *T*_1_ diagnostic, GVB-srDFT resembles HF-srDFT and is 0.3–0.4 eV less accurate than CAS-srDFT. In our data set, this occurs for the 2^1^ *A*_1_ state of furan and the 1^1^ *B*_2u_ state of s-tetrazine.

**Table 1:** Summary of Error Statistics (in eV) for Singlet Excitations: Mean Signed Deviation (MSD), Mean Absolute Deviation (MAD), Standard Deviation, and Maximum Absolute Error (MAX)^a^

method	MSD	MAD	std. dev.	MAX
LDA	–0.67	0.72	0.55	2.31
PBE	–0.69	0.74	0.58	2.49
CASSCF	0.48	0.55	0.53	2.46
GVB-PP	0.66	0.72	0.62	2.43
HF-srLDA	0.00	0.23	0.33	1.50
HF-srPBE	0.00	0.24	0.34	1.49
CAS-srLDA	–0.02	0.21	0.28	0.80
CAS-srPBE	–0.04	0.19	0.24	0.76
GVB-srLDA	–0.03	0.19	0.24	0.68
GVB-srPBE	–0.03	0.19	0.24	0.67
GVB-srPBE*	–0.04	0.19	0.24	0.67

a GVB-srPBE* refers to results obtained with reduced geminal space. All MC-srDFT calculations performed with μ = 0.4 bohr^–1^. Errors are given with respect to CC3 results.

**Figure 1:**
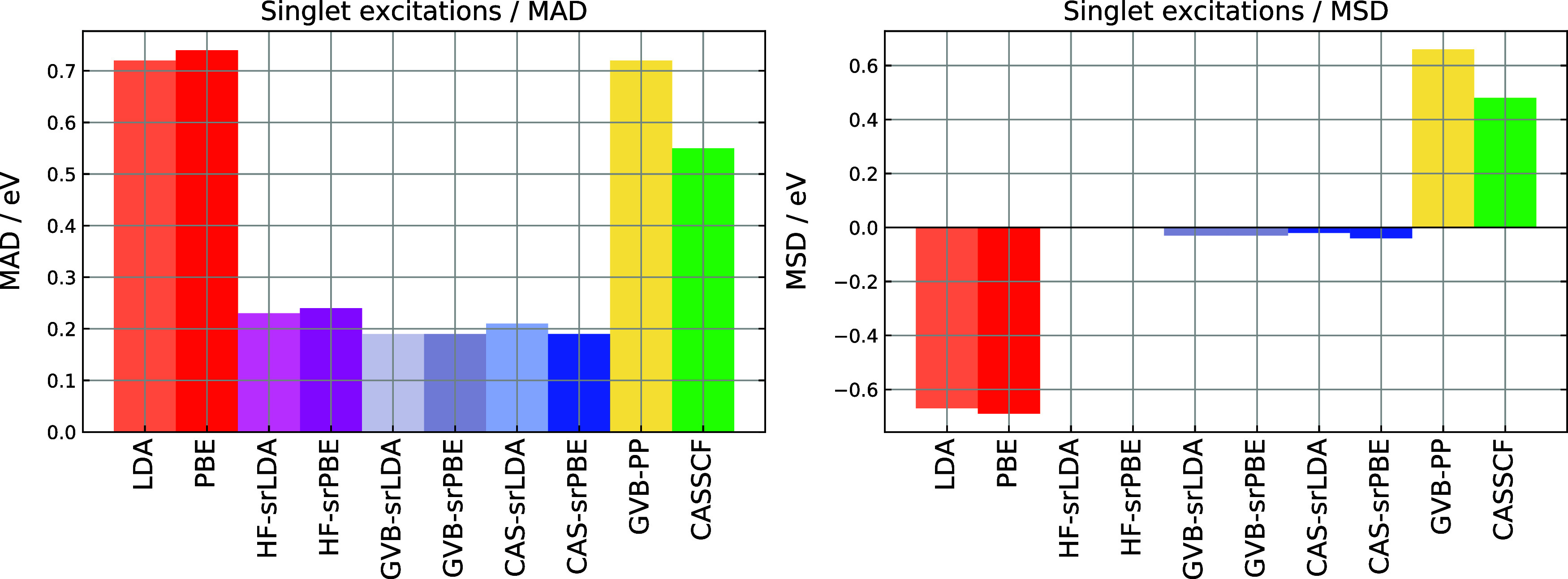
MAD (mean absolute deviation) and MSD (mean signed deviation) for singlet excitation energies obtained with DFT, srDFT, GVB-PP, and CASSCF models. The HF-, CAS-, and GVB-srDFT calculations used μ = 0.4 bohr^–1^.

In Figures 2 and 3, we compare error statistics for triplet excitations obtained using the full response (“full *E*[2]”) and the Tamm-Dancoff approximation, using CC3 results as the benchmark. The gTDA treatment proves beneficial for all methods. The largest improvement occurs for HF- and GVB-srDFT, where gTDA reduces the MAD by 0.2 eV. The effect is most pronounced for excitations below 3 eV, which are expected to suffer from near-triplet instabilities due to the presence of small elements in the Hessian matrix. Notably, the accuracy of GVB-srDFT triplet excitation energies using gTDA remains robust in the entire range studied, up to 10 eV (see Figure S6 in the Supporting Information).

**Figure 2:**
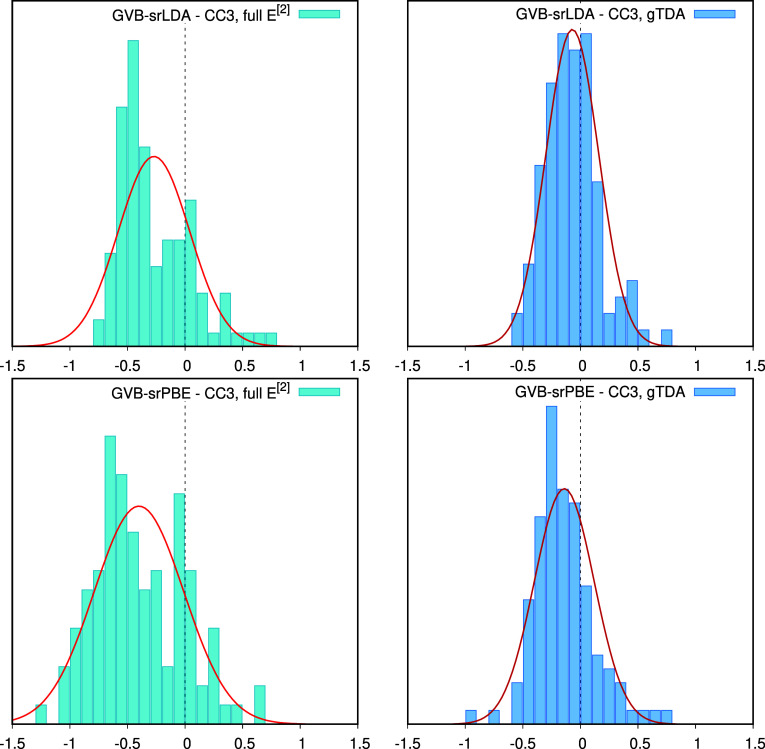
Deviations of GVB-srLDA (top) and GVB-srPBE (bottom) triplet excitations from the CC3 benchmark^74,75^ using full linear response (“full *E* ^[2]^, left) and gTDA (right). All GVB-srDFT calculations performed with μ = 0.4 bohr^–1^. The energy unit is eV.

**Figure 3:**
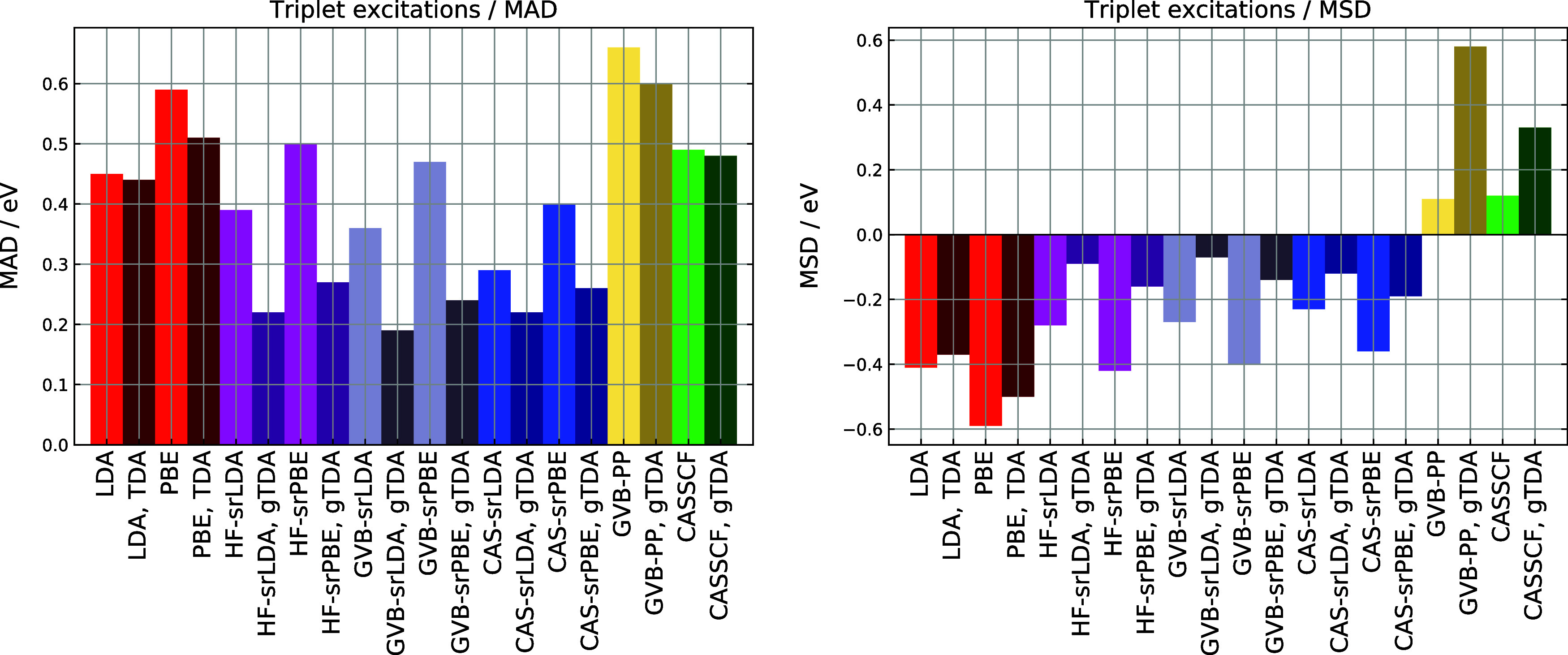
MAD (mean absolute deviation) and MSD (mean signed deviation) for triplet excitation energies obtained with DFT, srDFT, GVB-PP, and CASSCF models. HF-, CAS-, and GVB-srDFT calculations used the μ = 0.4 bohr^–1^ value. The darker color indicates that gTDA was applied.

Similar to the singlet response, GVB-PP systematically overestimates triplet excitations relative to the CC3 reference and is less accurate than CASSCF, with MAD values of 0.6 and 0.5 eV, respectively (see Table 2). Pure LDA and PBE underestimate the excitation energies to a comparable extent, the respective MSDs amounting to −0.4 and −0.6 eV. In MC-srDFT, the srLDA functional is slightly more accurate compared to srPBE, regardless of the long-range wave function. Range-separation effectively remedies the deficiencies of the GVB-PP model: GVB-srDFT performs on a par with CAS-srDFT, achieving MAD of 0.2 eV and a standard deviation of similar magnitude (see Table 2). Overall, GVB-srLDA and CAS-srLDA are the two best-performing methods. The 3^3^ *B*_2_ state of pyridine is the outlier in the set, as both GVB- and CAS-srLDA overestimate the CC3 value by as much as 0.7–0.8 eV. Here, GVB-srLDA­(gTDA) and CAS-srLDA­(gTDA) excitation energies of 8.06 and 8.03 eV, respectively, are much closer to the CASPT2 result^74^ in the same aug-cc-pVTZ basis (7.6 eV) than to the CC3 reference (7.29 eV).

**Table 2:** Summary of Error Statistics (in eV) for Triplet Excitations^a^

method	MSD	MAD	std. dev.	MAX
LDA	–0.37	0.44	0.45	1.37
PBE	–0.50	0.51	0.40	1.64
CASSCF	0.33	0.48	0.66	3.15
GVB-PP	0.62	0.64	0.64	3.30
HF-srLDA	–0.09	0.22	0.25	0.86
HF-srPBE	–0.16	0.27	0.27	0.77
CAS-srLDA	–0.12	0.21	0.22	0.74
CAS-srPBE	–0.19	0.26	0.24	0.73
GVB-srLDA	–0.05	0.19	0.23	0.77
GVB-srLDA*	–0.06	0.19	0.23	0.76
GVB-srPBE	–0.13	0.24	0.26	0.77
GVB-srPBE*	–0.15	0.25	0.26	0.74

a All methods employ the (generalized) Tamm-Dancoff approximation. GVB-srDFT* refers to results obtained with reduced geminal space. All MC-srDFT calculations performed with μ = 0.4 bohr^–1^. Errors are given with respect to CC3 results

A detailed inspection reveals that the largest deviations between GVB-srDFT and CAS-srDFT triplet excitations, ranging from 0.3 to 0.5 eV, occur for five systems characterized by increased static correlation:^65^ benzene (the 1^3^ *B*_1u_ and 1^3^ *B*_2u_ states), naphthalene (the 2^3^ *B*_1g_ and 3^3^ *A*_g_ states), *all-E*-octatetraene (the 1^3^ *B*_u_ state), pyridine (the 3^3^ *A*_1_ state), and s-tetrazine (the 1^3^ *B*_1u_ state). For these excitations, GVB-srDFT is closer to HF-srDFT. Considering the subset of 36 triplet excitations associated with these molecules, the differences between the srDFT approaches are reflected mainly in the error spread: GVB-srDFT improves over HF-srDFT, but remains slightly less accurate than CAS-srDFT, with standard deviations of 0.26, 0.30, and 0.22 eV, respectively (see Table S2 in the Supporting Information). For singlet excitations in the same subset, the differences between multi- and single-reference srDFT methods are even more pronounced. At the srPBE level, both GVB- and CAS-srPBE reduce the MAD by roughly 0.2 eV relative to HF-srPBE, while the standard deviation decreases from 0.5 eV (HF-srPBE) to 0.2 and 0.3 eV for GVB-srPBE and CAS-srPBE, respectively.

In general, MC-srDFT methods require smaller active spaces than their μ = *∞* limit, provided the range-separation parameter is sufficiently small to allow short-range correlation effects to be captured by the srDFT functional.^46^ For CAS-srDFT, the value of μ = 0.4 has been shown to meet this criterion.^54,65,80^ To verify if the same choice of μ allows for smaller active spaces for geminal-based wave functions, we compared excitation energies from GVB-srDFT (μ = 0.4) calculations performed either with the full-valence GVB-PP active space or with reduced number of geminals. The reduced space was selected based on MP2-srDFT occupation numbers. The differences between individual excitation energies from the two active spaces do not exceed 0.1 eV, and the corresponding error statistics are virtually identical (see GVB-srDFT* entries in Tables 1 and 2, as well as Table S3 in the Supporting Information). Thus, using the full-valence active space offers no clear advantage, and restricting geminals to the strongly correlated orbitals is recommended, consistent with the typical approach in CAS-srDFT.

### 4.2 SSCCs for Single-Reference Molecules

In this section, we assess the performance of GVB-PP and GVB-srDFT methods in computing indirect spin–spin coupling constants. Since our reference data set consists of small molecules of a single-reference character, we report errors relative to the CC3 benchmark values.

Overall, couplings from GVB-PP are less accurate than the CASSCF ones, with largest deviations between the two models occurring for couplings involving fluorine. Figure 4 (top panels) compares the errors of GVB-PP and CASSCF for the FC contributions to the coupling tensor (results for PSO and SD terms are given in Supporting Information, see Figures S9 and S10). The FC contributions are of a particularly poor quality compared to the remaining contributions computed at the GVB-PP level: GVB-PP deviates from CASSCF not only for fluorine, but also for *J*_CC_ (FCCH, FCCF) and ^1^ *J*_HC_ (HCCH, HFCO, FCCH) couplings. Invoking the gTDA approximation worsens the agreement between GVB-PP and CASSCF even further. A boxplot representation of errors reveals that gTDA reduces the GVB-PP error for the triplet SD term, yet the quality of the FC and PSO contributions deteriorates (see Figure S11 in the Supporting Information). In fact, the failure of the gTDA model for the FC contribution persist across all methods, including KS-DFT and MC-srDFT approaches, consistently with earlier findings in the literature.^54,81^

**Figure 4:**
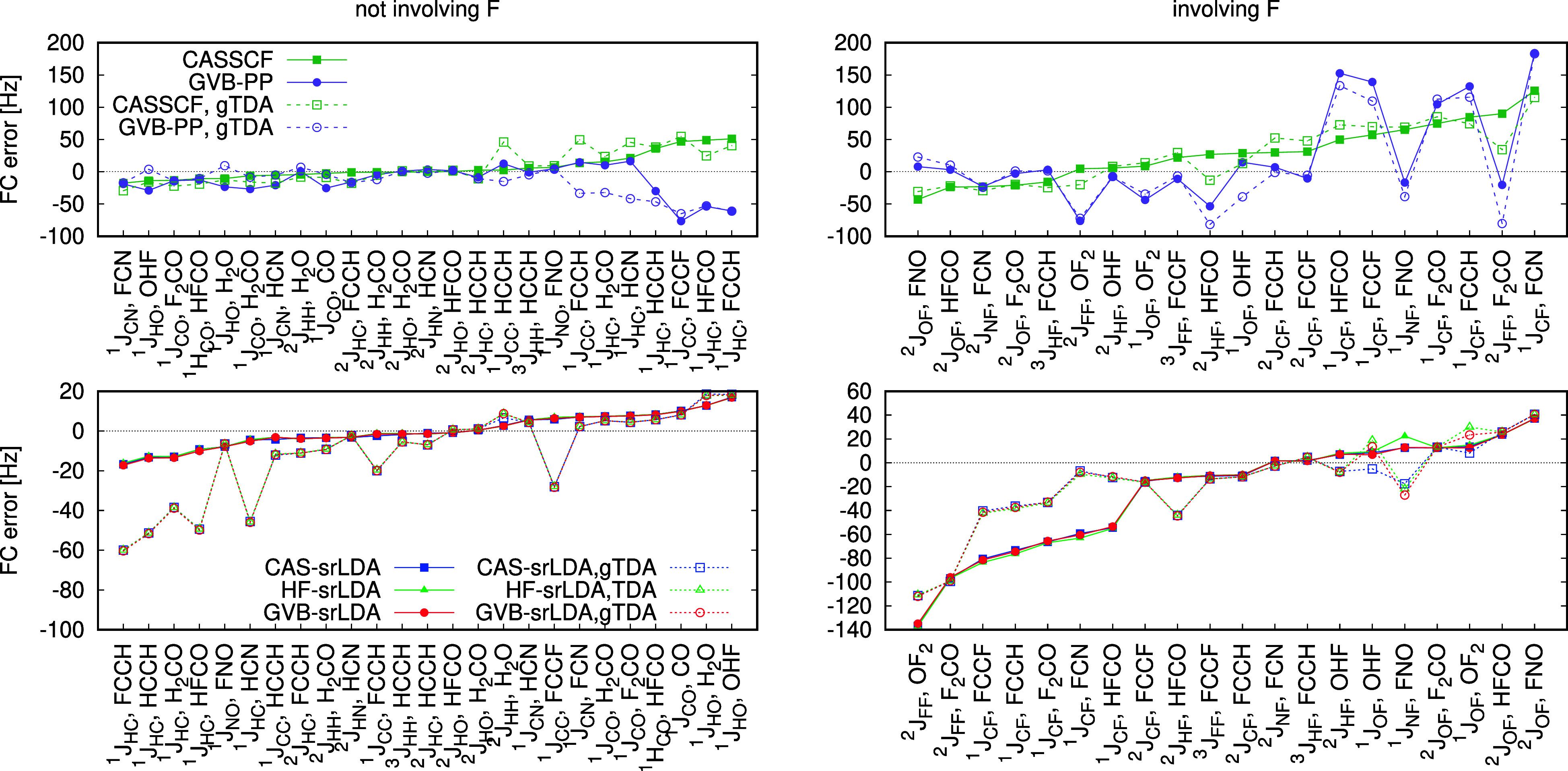
Errors relative to CC3 in the FC contributions to the SSCCs obtained in the “full E[2]” and gTDA approaches, sorted from the most negative to most positive error in CASSCF/CASsrDFT calculations. Lines are drawn to guide to eye.

Adding dynamic correlation via the short-range density functional significantly improves the accuracy of GVB-based couplings. As shown in the bottom panels of Figure 4, the GVB-srLDA and CAS-srLDA results are nearly identical. In the “full E^[2]^” approach, the deviations do not exceed 2 Hz. When gTDA is employed, the differences between CAS-srLDA and GVB-srLDA become more pronounced: for three couplings involving fluorine the methods deviate by 10–20 Hz [^1^ *J*_NF_ (FNO) and ^1^ *J*_OF_ (OHF, OF_2_)]. In these cases, GVB-srLDA is closer to HF-srLDA. Overall, gTDA improves SD and PSO terms irrespective of the long-range wave function in the MC-srDFT model. For FC components the picture is different: gTDA is beneficial only for FC couplings involving fluorine, while the remaining types are more accurate when gTDA is not applied (see also Figures S11 and S12 in the Supporting Information). Irrespective of gTDA, the most problematic FC contributions are of the ^2^ *J*_FF_ and ^1^ *J*_CF_ types; their exclusion markedly improves MC-srDFT accuracy (cf. Figure S12 in the Supporting Information). Finally, since srLDA and srPBE perform similarly, the cheaper srLDA model is recommended.

### 4.3 Transition Metal MF_6_^–*z*^ Complexes

In ref 54, Kjellgren and Jensen analyzed SSCCs of the ^1^ *J*_MF_ and ^1^ *J*_MC_ types in transition metal complexes stabilized by either carbon monoxide [M­(CO)_*y*_^–*z*^] or fluorine [MF_6_^–*z*^] ligands. In this work, we focus on the most challenging system from that test set: VF_6_^–^. This is the only complex in the group where both FC and PSO terms contribute to the coupling by comparable magnitude. Since these terms have opposite signs, getting the correct cancellation between them becomes particularly challenging. As reported in ref 54, satisfactory accuracy for carbonyl complexes is already achieved at the Kohn–Sham DFT level, and introducing range separation brings little or no improvement. Therefore, these complexes are not included in the present study. Results for the remaining fluorine-containing systems from ref 54 (ScF_6_^–3^ and TiF_6_^–2^) are provided in the Supporting Information. Reference data are taken from ref 82. Each MC-srDFT method is applied with four variants for solving the linear response problem: full response, gTDA, or gTDA applied to only the singlet or only the triplet part. Following ref 54, the range separation parameter μ is set to 1.0 bohr^–1^ for all transition-metal systems. All CAS-srDFT results were obtained using a CAS­(10,10) active space, which includes the full d-shell. Similar to the single-reference systems discussed in the previous section, srLDA and srPBE functionals yield SSCCs of comparable quality (see also Table S4 in the Supporting Information), and only srLDA results are discussed below.

In Figure 5 we show the ^1^ *J*_VF_ couplings in the VF_6_^–^ complex. The application of gTDA has a large impact on the couplings, with a significant effect both in the triplet (FC, SD) and singlet (PSO) contributions. This stays in contrast to ScF_6_^–3^ and TiF_6_^–2^, where the FC term dominates, so that invoking gTDA in the singlet response equations has little effect (see Figures S14 and S15 in the Supporting Information). As shown in Figure 5, MC-srDFT methods clearly outperform both DFT and pure wave function (GVB-PP and CASSCF) approaches. In agreement with ref 54, only one variant of MC-srDFT response provides accurate results, i.e., using gTDA for singlet components and solving the full response for the triplet ones. This approach yields similar accuracy regardless of the long-range wave function type, when combined with the srLDA functional. The relatively small influence of nondynamical correlation is consistent with the large natural occupation numbers, all exceeding 1.98 at the GVB-PP level. For ScF_6_^–3^ and TiF_6_^–2^, MC-srDFT again clearly outperforms DFT and markedly improves upon the pure CASSCF and GVB-PP methods, with best results obtained using either full response or gTDA in the singlet part (see Figures S14 and S15 in the Supporting Information).

**Figure 5:**
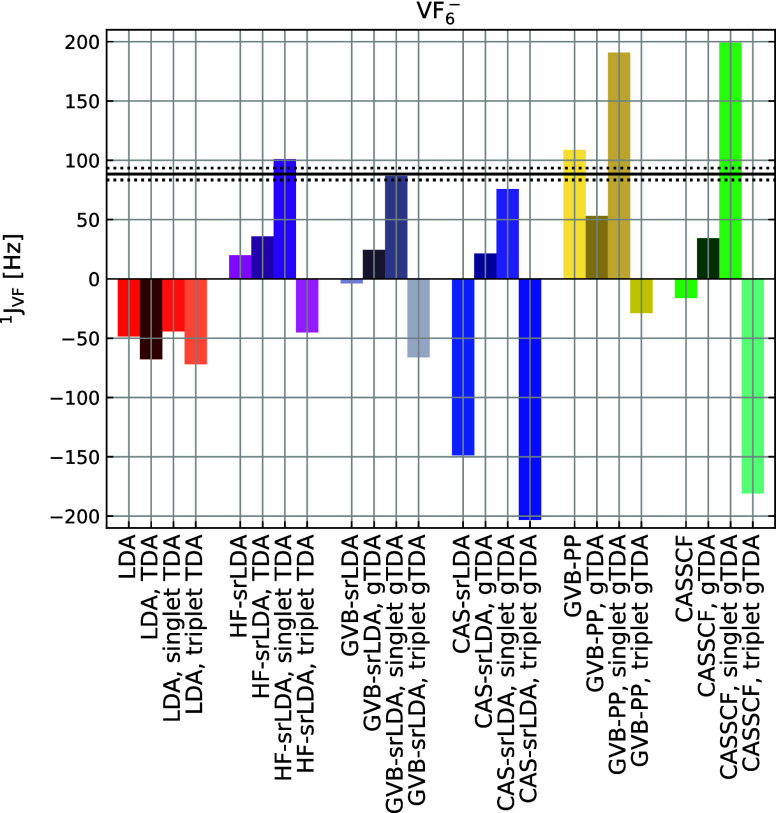
Calculated SSCCs for the VF_6_^–1^ complex. All the srDFT calculations are with μ = 1.0 bohr^–1^. The black dashed lines represent the reference coupling ±5 Hz. For each method, four results are reported: full *E*[2], full gTDA, only gTDA in singlet response, and only gTDA in triplet response.

Overall, the largest improvement of MC-srDFT over DFT comes from mitigating self-interaction error through range separation. The effect of accounting for multireference character is considerably smaller, but still noticeable: GVB-srDFT and CAS-srDFT offer similar accuracy and are systematically better than HF-srDFT.

## 5 Conclusions

In this work we presented a method for treating dynamic correlation in strongly orthogonal geminal wave function theory, applicable to both ground and excited states. The approach is formulated in the framework of multiconfiguration range-separated DFT theory^45,46^ and its time-dependent extension.^39,83^ It combines long-range wave function of the GVB-PP type with short-range functionals from the LDA (srLDA^71^) and GGA (srPBE^72^) rungs of the Jacob’s ladder. GVB-srDFT wave function optimization is performed employing the restricted step second-order algorithm previously developed for CAS-srDFT^59^ as an extension of the original MCSCF implementation.^55,57,60^ For the initial geminal guess we use the black-box protocol first introduced for GVB-PP and APSG wave functions.^40^ Excited states of singlet and triplet character are accessed via linear-response GVB-srDFT theory, derived for singlet reference. Linear response equations are solved using a direct iterative algorithm^63,84^ as implemented in the Dalton^58,69^ program.

We focused on determining whether GVB-srDFT adequately describes excited states through linear response. To this end, we assessed its accuracy for both singlet and triplet excitation energies on a diverse set of molecules from benchmark data sets of Loos et al.^75^ and Schreiber et al.^73^ for which CC3 reference results are available. These small and medium-sized systems also allow direct comparison with a more accurate member of the MC-srDFT family, namely the CAS-srDFT method. GVB-srDFT offers a substantial improvement over the pure GVB-PP model: for singlet excitation energies, the MAD is reduced from 0.7 eV (GVB-PP) to 0.2 eV (GVB-srDFT). For triplet excitations, the corresponding MADs amount to 0.6 and 0.2 eV, respectively, when computed using the generalized Tamm-Dancoff approximation. When gTDA is not invoked, the performance deteriorates, with MADs increasing to 0.4 and 0.5 eV at the GVB-srLDA and GVB-srPBE levels, respectively. The more expensive srPBE functional offers no advantage over srLDA; in fact, the latter is systematically more accurate for triplet response and is the recommended choice.^65^

Importantly, GVB-srDFT closely matches the accuracy of CAS-srDFT, which is similar to that of CC2 and ADC(2) methods.^65,75^ On a subset of systems with enhanced static correlation,^65^ GVB-srDFT and CAS-srDFT perform on par for singlet excitations, both reducing the MAD by 0.2 eV relative to HF-srDFT. However, for triplet excitations in the same subset, GVB-srDFT remains closer to HF-srDFT, with a larger spread of errors than CAS-srDFT, as indicated by standard deviations of 0.3 eV (GVB-srDFT) and 0.2 eV (CAS-srDFT). A key limitation of both GVB-PP and GVB-srDFT is their inability to describe certain doubly excited states (see the discussion in refs 39, 40 ). This leads to larger errors for excitations with significant contributions from doubly excited determinants—for example, the 2 ^1^ *A*_1_ state of furan, where GVB-srLDA is approximately 0.3 eV less accurate than CAS-srLDA.

Indirect spin–spin couplings serve as a sensitive probe of the quality of linear response functions, as they depend on both singlet and triplet response components. We verified the performance of GVB-srDFT for SSCCs by comparison with CC3 benchmarks using a data set of small single-reference molecules,^78^ as well as the challenging transition metal complex VF_6_^–^.^54^ In the molecular data set, couplings computed with pure GVB-PP are clearly less accurate than those from CASSCF. In contrast, GVB-srDFT and CAS-srDFT results match closely. Since nondynamical correlation effects are negligible in this set, HF-srDFT is as accurate as both MC-srDFT models. For couplings not involving fluorine, range separated approaches improve upon both the pure KS-DFT and wave function limits, offering a quantitative description. However, for the most problematic cases, that is, couplings involving fluorine, range separation alone does not compensate for the deficiencies of the short-range functionals. In consequence, none of the MC-srDFT variants proves satisfactory for couplings with fluorine, with ^1^ *J*_FF_, ^1^ *J*_OF_, and ^1^ *J*_CF_ presenting the greatest challenge.

Our study supports the recommendation that, within the MC-srDFT framework, gTDA should be applied only to the singlet response when calculating SSCCs. As observed in ref 54, gTDA systematically improves the singlet PSO contributions, but is detrimental to FC triplet contributions. This indicates that low-lying triplet excitations, which are better described with gTDA, are not critical for getting the correct FC terms. While gTDA also benefits the SD terms, these are generally small in magnitude.

For the vanadium–fluorine coupling in the VF_6_^–^ complex, use of TDA/gTDA is crucial to achieving the correct cancellation between the negative PSO and positive FC components. Across three isoelectronic MF_6_^–*z*^ complexes (ScF_6_^–3^, TiF_6_^–2^, and VF_6_^–^), GVB-srLDA consistently outperforms HF-srLDA and is comparable to, or slightly better than, CAS-srLDA.

One of the advantages of MC-srDFT methods is that they provide accurate results with smaller active spaces than required in full wave function approaches. We confirmed that this also applies to GVB-srDFT: singlet and triplet excitation energies computed with a reduced number of geminals deviate by no more than 0.1 eV from those obtained using a full-valence active space, and the associated error statistics are virtually identical. To define the reduced active space, we selected geminals based on MP2-srDFT natural occupation numbers. Finally, we recommend using the standard value of μ = 0.4 bohr^–1^ for GVB-srDFT, as it provides excellent accuracy for excitation energies and SSCCs in organic molecules. In contrast, reliable prediction of SSCCs in transition metal complexes requires a larger value, μ = 1.0 bohr^–1^, likely due to medium-range nondynamical correlation effects involving the metal *d*-shell.^54^

To summarize, our results demonstrate that TD-GVB-srDFT provides a reliable description of excited states. The method is especially promising for systems in which the active space required for CAS-srDFT becomes prohibitively large. Future extensions employing on-top pair densities in the short-range functionals should enable a correct description of spin degeneracies and significant reduction of self-interaction error.^50,85^ Moreover, GVB-srDFT linear response can be adapted to describe noncovalent interactions in systems with significant nondynamical correlation, within the framework of multiconfigurational symmetry-adapted perturbation theory, SAPT­(MC).^86,87^ Ongoing work in our groups is focused on developing this approach.

## Supplementary Material





## Data Availability

The raw data are available in the Zenodo repository at 10.5281/zenodo.17052974.

## Notes

Additional details on GVB-srDFT linear response equations; boxplot representation of errors for singlet/triplet excitations and SSCCs; and visualization of VF_6_^–1^ geminals (PDF)
Singlet and triplet excitation energies and SSCCs for systems of Sections 4.2 and 4.3 (XLSX)
