# Supplementary material for: Time-Dependent Multiconfigurational Short-Range Density Functional Theory with Generalized Valence Bond Wave Functions
Source: J Phys Chem A. 2025 Sep 29;129(40):9464–76. doi: 10.1021/acs.jpca.5c04699 (PMC12516726; doi:10.1021/acs.jpca.5c04699)
Supplement: Supplementary file 1 [file jp5c04699_si_001.pdf]

Supporting Information: Time-Dependent Multiconfigurational Short-Range Density Functional Theory with Generalized Valence Bond Wave Functions

Michał Hapka*,† and Hans Jørgen Aa. Jensen*,‡

† *University of Warsaw, Faculty of Chemistry, ul. L. Pasteura 1, 02-093 Warsaw, Poland*

‡ *Department of Physics, Chemistry and Pharmacy, University of Southern Denmark, Campusvej 55, Odense M, Denmark*

E-mail: michal.hapka@uw.edu.pl; hjj@sdu.dk

Contents

1 Details on GVB-srDFT linear response **S-3**

2 Additional Results **S-5**

2.1 Singlet and Triplet Excitation Energies S-11

2.2 SSCCs: comparison to CC3 benchmark values S-16

2.3 Transition metal complexes with F ligands, MF_6^{-z} S-20

1 Details on GVB-srDFT linear response

For the singlet response, the relevant srDFT contributions to geminal σ-vectors read

$$ {}^{Z}\sigma_i^{g,\mathrm{sr}} = \sum_j \frac{\partial^2 E_{\mathrm{xc}}^{\mathrm{sr}}}{\partial c_j \partial c_i^*} b_j^g = 2c_i V_{\mathrm{srxc},ii}^{C,[1g]} + 2b_i^g V_{\mathrm{srxc},ii}^{C} \tag{S.1} $$

$$ {}^{Y}\sigma_i^{g,\mathrm{sr}} = \sum_j \frac{\partial^2 E_{\mathrm{xc}}^{\mathrm{sr}}}{\partial c_j \partial c_i} b_j^g = -2c_i V_{\mathrm{srxc},ii}^{C,[1g]} - 2b_i^g V_{\mathrm{srxc},ii}^{C} \tag{S.2} $$

where in Eq. (S.2) we have used that we have real geminals. We have also used that the geminal trial vectors always only have a Z-part in our implementation. The geminal contributions to orbital σ-vectors are computed as

$$ \begin{aligned} {}^{Z}\sigma_{\mathrm{srxc},i}^{o} &= \sum_{r \neq s} \frac{\partial^2 E_{xc}^{sr}}{\partial c_i \partial \bar{\kappa}_{rs}} b_{rs}^o = 2c_i \left(V_{\mathrm{srxc},ii}^{C,[1o]} + \tilde{V}_{\mathrm{srxc},ii}^{C} \right) \\ {}^{Y}\sigma_{\mathrm{srxc},i}^{o} &= \sum_{r \neq s} \frac{\partial^2 E_{xc}^{sr}}{\partial c_i^* \partial \bar{\kappa}_{rs}} b_{rs}^o = -2c_i \left(V_{\mathrm{srxc},ii}^{C,[1o]} + \tilde{V}_{\mathrm{srxc},ii}^{C} \right) \end{aligned} \tag{S.3} $$

Note the unrestricted summation; b_{rs}^o is in the Z-vector for $r > s$ and in the Y-vector for $r < s$. The long-range parts of the σ-vectors take the following form:

$$ \begin{aligned} {}^{Z}\sigma_i^{o,\mathrm{lr}} &= \sum_{r \neq s} \frac{\partial^2 E^{lr}}{\partial c_i \partial \bar{\kappa}_{rs}} b_{rs}^o = 2c_i \tilde{F}_{ii}^D + \sum_{j \in I_i} c_j \widetilde{\langle ii|jj \rangle} - \sum_{j \notin I_i} 2c_i c_j^2 \left(2\widetilde{\langle ij|ij \rangle} - \widetilde{\langle ij|ji \rangle} \right) \\ {}^{Y}\sigma_i^{o,\mathrm{lr}} &= \sum_{r \neq s} \frac{\partial^2 E^{lr}}{\partial c_i^* \partial \bar{\kappa}_{rs}} b_{rs}^o = 2c_i \tilde{F}_{ii}^D + \sum_{j \in I_i} c_j \widetilde{\langle jj|ii \rangle} - \sum_{j \notin I_i} 2c_i c_j^2 \left(2\widetilde{\langle ij|ij \rangle} - \widetilde{\langle ij|ji \rangle} \right) \end{aligned} \tag{S.4} $$

where the only difference between Z and Y components is in the second term on the right-hand-side, as $\widetilde{\langle ii|jj \rangle} \neq \widetilde{\langle jj|ii \rangle}$ when $b_{rs}^o \neq -b_{sr}^o$. The one-index transformed Fock matrix elements read

$$ \tilde{F}_{ij}^D = \tilde{h}_{ij} + \sum_k D_{kk}^C \left(2\widetilde{\langle ik|jk \rangle} - \widetilde{\langle ik|kj \rangle} \right). \tag{S.5} $$

Finally, the orbital contributions for the singlet response read

$$\sigma_{pq}^{C,o} = \left\langle \Psi_{\text{GVB}}^{\text{lr}} \middle| \left[\hat{E}_{pq}, \hat{\tilde{H}}^{\text{lr}} \right] \middle| \Psi_{\text{GVB}}^{\text{lr}} \right\rangle \tag{S.6}$$

$$\sigma_{\text{srxc},pq}^{C,o} = \left\langle \Psi_{\text{GVB}}^{\text{lr}} \right| \left[\hat{E}_{pq}, \hat{V}_{\text{srxc}}^{C,[1o]} + \hat{\tilde{V}}_{\text{srxc}}^{C} \right] \left| \Psi_{\text{GVB}}^{\text{lr}} \right\rangle . \tag{S.7}$$

2 Additional Results

Table S1: The active space used in singlet/triplet excitation calculations. *inact* and *ngem* denote the number of inactive orbitals and geminals, respectively.

	GVB-srDFT		GVB-srDFT*	
	GVB-PP act. space		reduced act. space	
	inact	ngem	inact	ngem
Acetaldehyde	3	9	10	2
Acetamide	4	12	14	2
Acetone	4	12	14	2
Acetylene	2	5	5	2
Ammonia	1	4	2	3
Benzene	6	15	19	2
Carbonmonooxide	2	5	5	2
Cyclopentadiene	5	13	16	2
Cyclopropene	3	8	8	3
Diazomethane	3	8	9	2
Dinitrogen	2	5	5	2
E-Butadiene	4	11	13	2
Ethylene	2	6	6	2
Formaldehyde	2	6	6	2
Formamide	3	9	10	2
Furan	5	13	16	2
Hexatriene	6	16	19	3
Hydrogensulfide	1	8	6	3
Imidazole	5	13	15	3
Ketene	3	8	9	2
Methanamine	2	6	6	2
Naphthalene	10	24	30	4
Nitrosomethane	3	9	10	2
Norbornadiene	7	18	23	2
Octatetraene	8	21	25	4
p-Benzoquinone	8	20	24	4
Propanamide	5	15	17	3
Pyridine	6	15	19	2
Pyrrole	5	13	16	2
s-Tetrazine	6	15	19	2
Streptocyanine-C1	3	9	10	2
Thioformaldehyde	2	10	9	3
Water	1	4	3	2

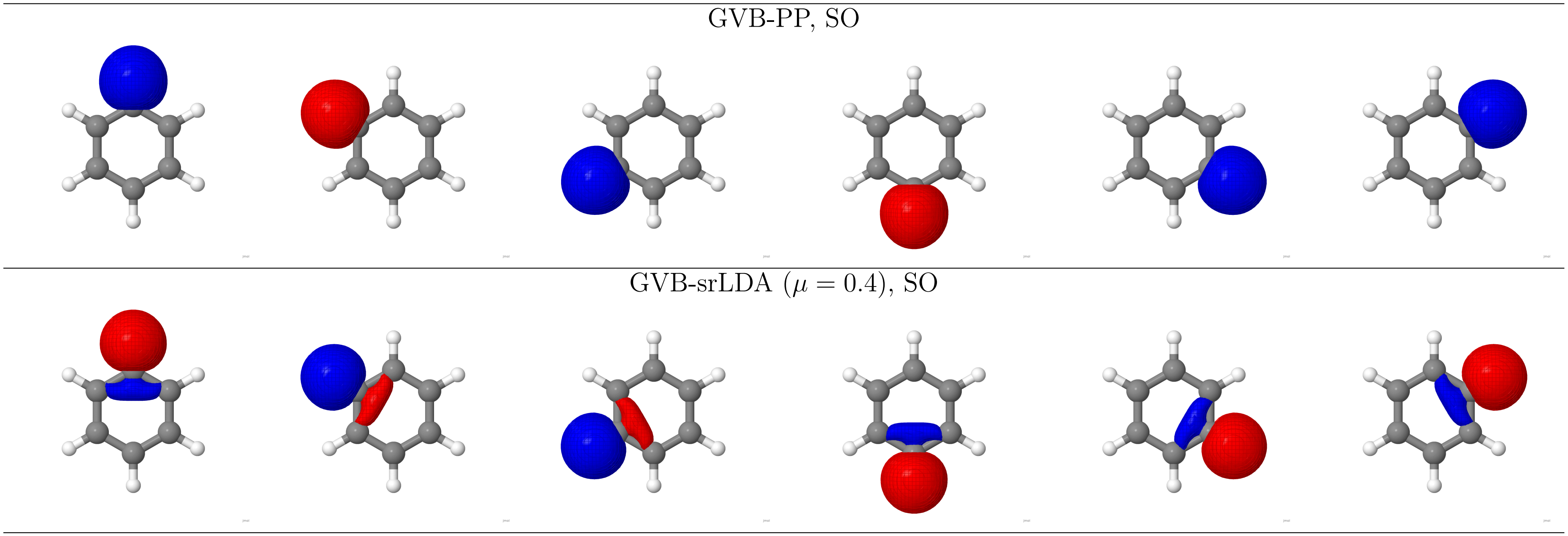

Figure S1: Benzene: the C-H geminals from GVB-PP (top) and GVB-srLDA (bottom) calculations (only **SO components** shown, Pipek-Mezey localization). TZVP basis set.

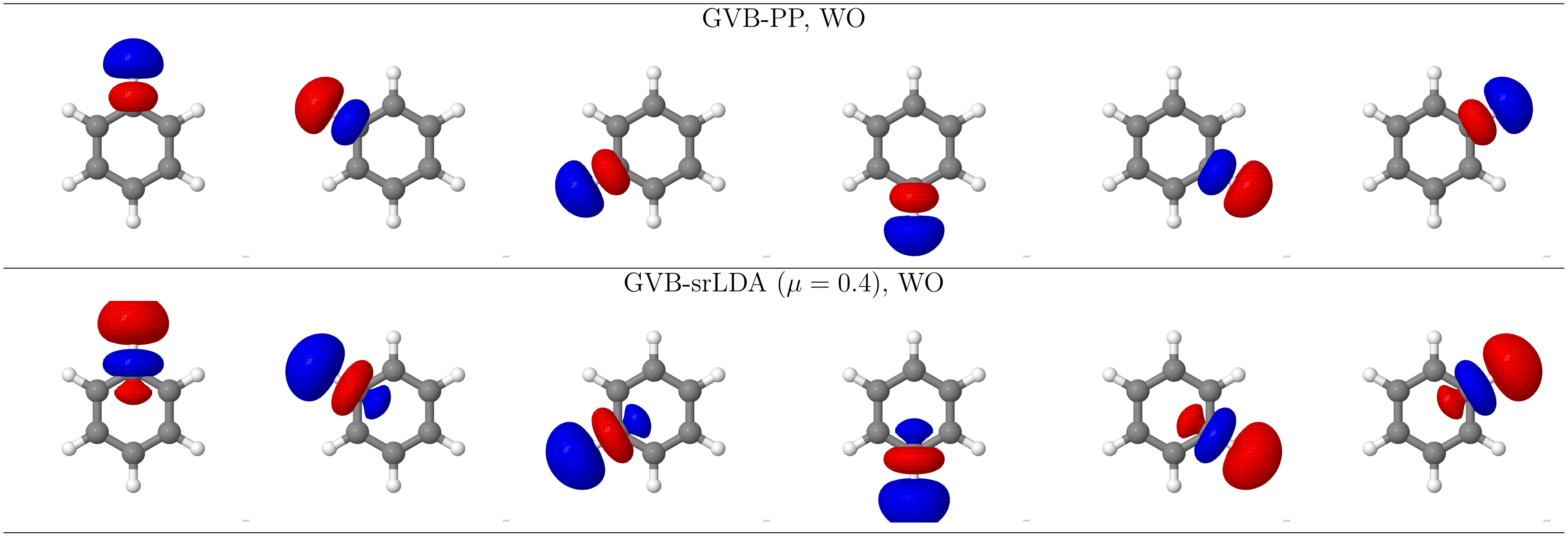

Figure S2: Benzene: the C-H geminals from GVB-PP (top) and GVB-srLDA (bottom) calculations (only **WO components** shown, Pipek-Mezey localization). TZVP basis set.

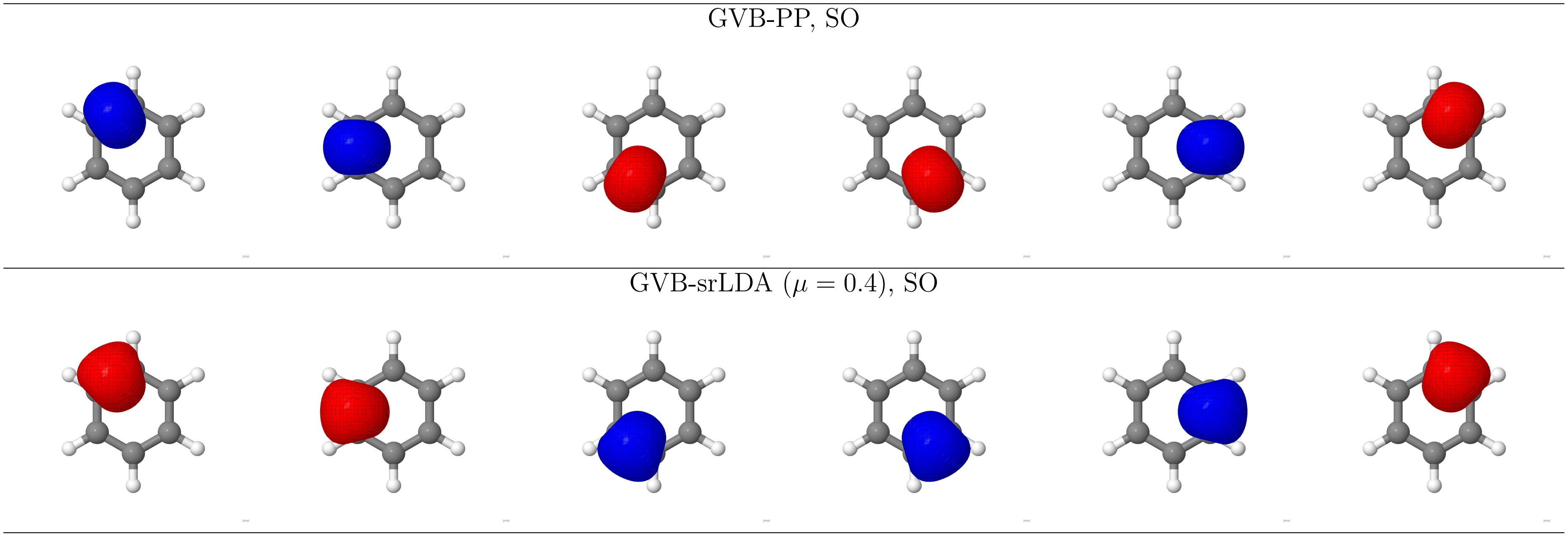

Figure S3: Benzene: the σ C-C geminals from GVB-PP (top) and GVB-srLDA (bottom) calculations (only **SO components** shown, Pipek-Mezey localization). TZVP basis set.

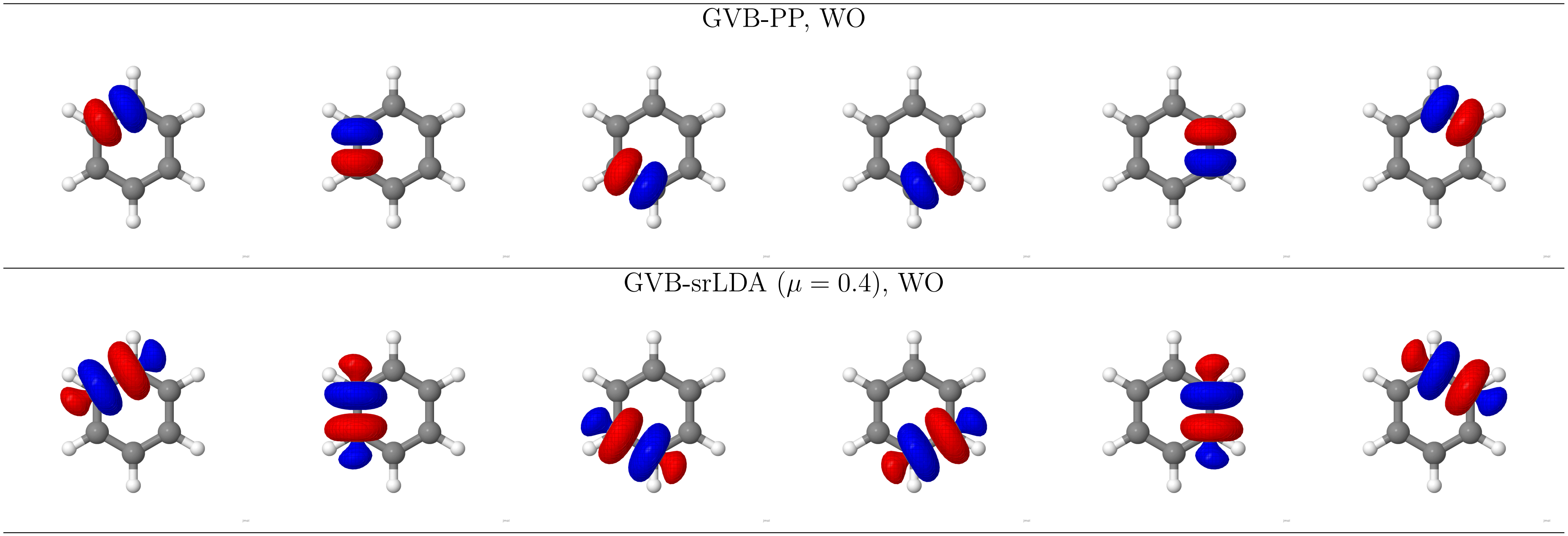

Figure S4: Benzene: the σ C-C geminals from GVB-PP (top) and GVB-srLDA (bottom) calculations (only **WO components** shown, Pipek-Mezey localization). TZVP basis set.

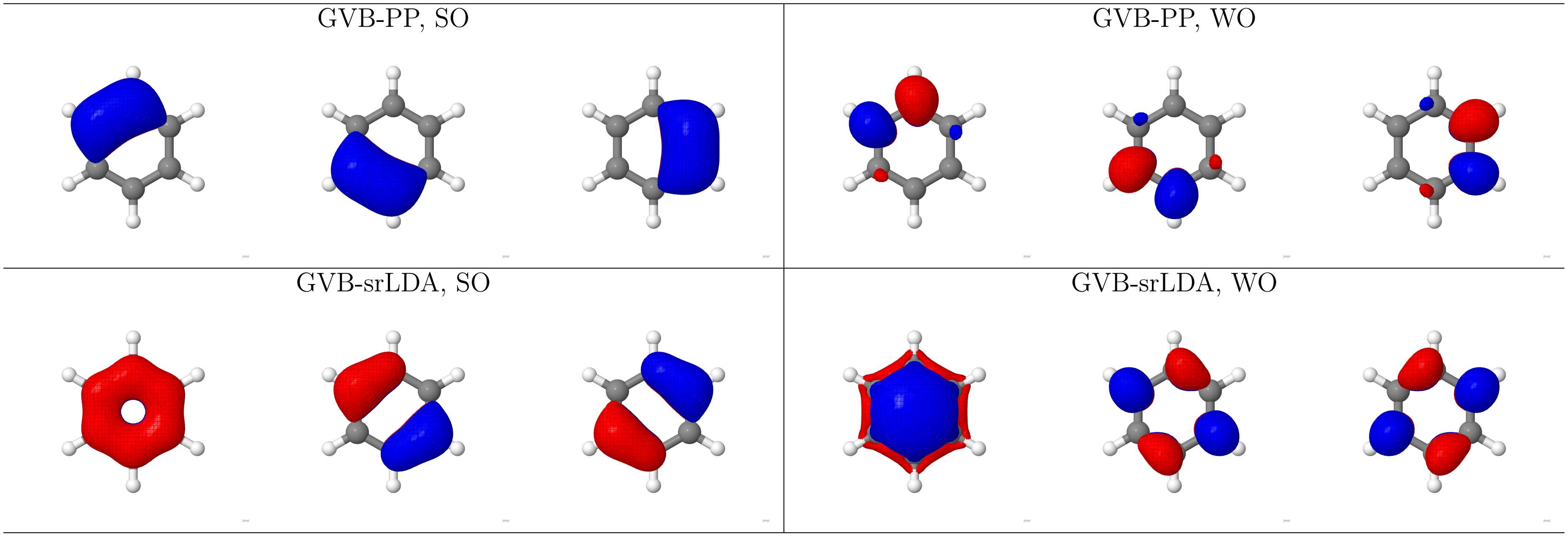

Figure S5: Benzene: the π C-C geminals from GVB-PP (top) and GVB-srLDA (bottom) calculations (**left**: SO components, **right:** WO components). Pipek-Mezey localization. TZVP basis set.

2.1 Singlet and Triplet Excitation Energies

Table S2: Summary of error statistics (in eV) for the subset of 27 singlet and 36 triplet excitations from **5 molecules with increased static correlation** (benzene, naphthalene, *all-E*-octatetraene, pyridine, s-tetrazine). For triplet excitations all methods employ the (generalized) Tamm-Dancoff approximation. All MC-srDFT calculations performed with $\mu = 0.4$. Errors are given with respect to CC3 results.

	Singlet excitations				Triplet excitations			
method	MSD	MAD	std dev	MAX	MSD	MAD	std dev	MAX
CASSCF	0.65	0.82	0.81	2.56	0.54	0.71	0.84	1.00
GVB-PP	1.03	1.08	0.72	2.22	0.88	0.92	0.85	3.30
HF-srLDA	0.17	0.33	0.45	1.50	0.05	0.22	0.30	0.86
HF-srPBE	0.20	0.38	0.50	1.49	0.00	0.24	0.31	0.75
CAS-srLDA	0.05	0.21	0.27	0.62	0.00	0.17	0.22	0.74
CAS-srPBE	0.06	0.21	0.27	0.64	−0.04	0.18	0.23	0.64
GVB-srLDA	0.03	0.19	0.26	0.64	0.07	0.20	0.26	0.77
GVB-srPBE	0.05	0.21	0.27	0.64	0.02	0.21	0.29	0.77

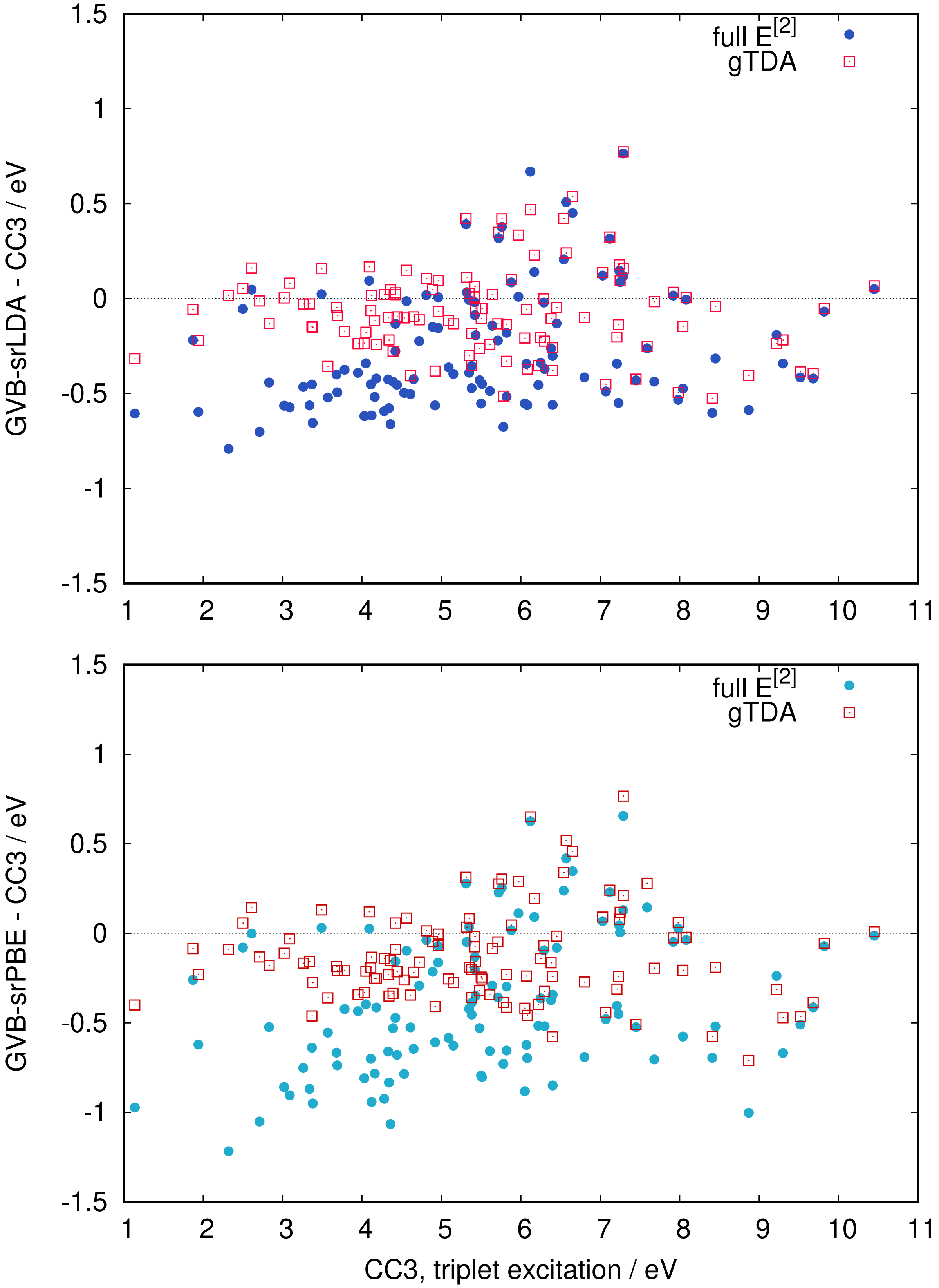

Figure S6: Deviations of GVB-srDFT triplet excitations from CC3 benchmark: GVB-srLDA (top) and GVB-srPBE (bottom). Basis set is aug-cc-pVTZ.

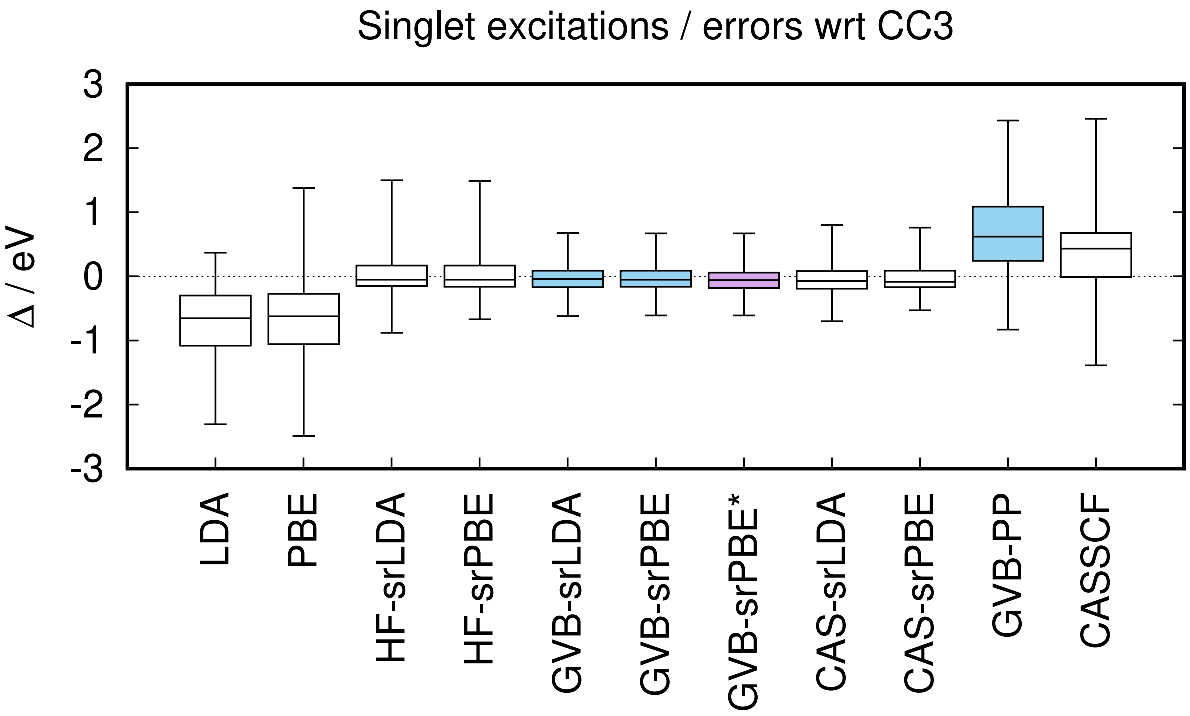

Figure S7: Box plots of errors (Δ, in eV) in singlet excitations. CAS- and GVB-srDFT methods used $\mu = 0.4$ bohr^{-1}. GVB-PP and GVB-srDFT results are marked in blue. GVB-srPBE* refers to results obtained using smaller active space, see Table S1 (marked in violet).

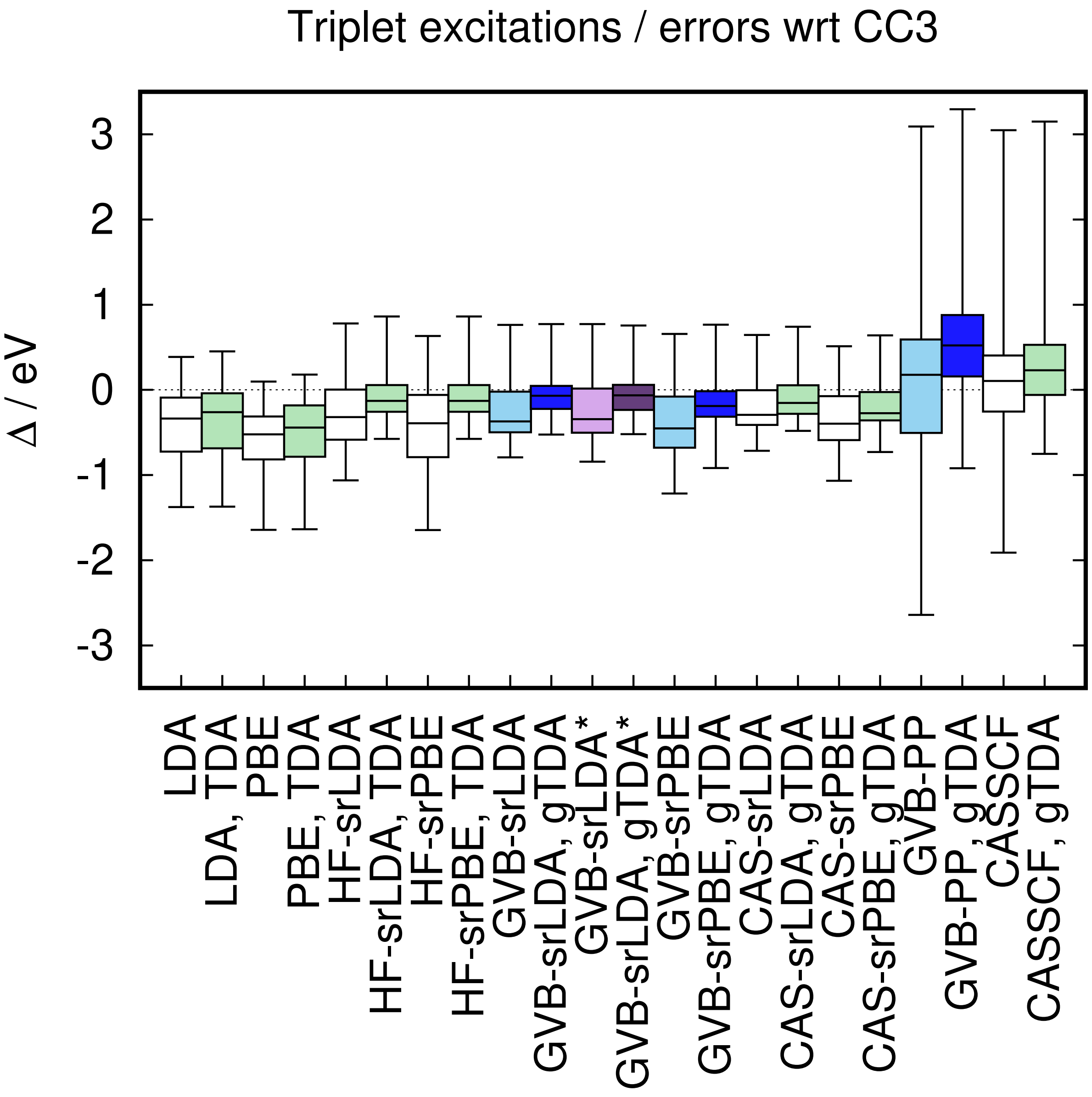

Figure S8: Box plots of errors (Δ, in eV) in triplet excitations. CAS- and GVB-srDFT methods used $\mu = 0.4$ bohr^{-1}. GVB-PP and GVB-srDFT results are marked in blue. GVB-srLDA* refers to results obtained using smaller active space, see Table S1 (marked in violet). The darker colors indicate that the Tamm-Dancoff approximation has been used.

Table S3: Statistics for triplet excitations: mean signed deviation (MSD), mean absolute deviation (MAD), standard deviation and maximum absolute error (MAX). Energy unit is eV. All MC-srDFT calculations performed with $\mu = 0.4$. The data set consists of 111 excitations. GVBsrLDA* refers to results obtained with reduced active space (see Table S1). For GVB-PP, four excitations could not be identified [benzene (1 ^{3}A$_g$), imidazole (2 ^{3}A$''$), napthalene (2 ^{3}B$_{3g}$, 3 ^{3}B$_{3g}$)].

method	MSD	MAD	std. dev.	MAX
LDA	-0.41	0.45	0.42	1.38
LDA, TDA	-0.37	0.44	0.45	1.37
PBE	-0.59	0.59	0.35	1.64
PBE, TDA	-0.50	0.51	0.40	1.64
HF-srLDA	-0.28	0.39	0.37	1.06
HF-srLDA,TDA	-0.09	0.22	0.25	0.86
HF-srPBE	-0.42	0.50	0.46	1.64
HF-srPBE, TDA	-0.16	0.27	0.27	0.77
CAS-srLDA	-0.23	0.29	0.26	0.72
CAS-srLDA, TDA	-0.12	0.21	0.22	0.74
CAS-srPBE	-0.36	0.40	0.33	1.07
CAS-srPBE, TDA	-0.19	0.26	0.24	0.73
CASSCF	0.12	0.49	0.72	3.05
CASSCF, TDA	0.33	0.48	0.66	3.15
GVB-PP	0.15	0.70	0.92	3.09
GVB-PP, TDA	0.62	0.64	0.64	3.30
GVBsrLDA	-0.25	0.35	0.32	0.79
GVBsrLDA, TDA	-0.05	0.19	0.23	0.77
GVBsrLDA*	-0.25	0.35	0.33	0.84
GVBsrLDA, TDA*	-0.06	0.19	0.23	0.76
GVBsrPBE	-0.39	0.46	0.39	1.22
GVBsrPBE, TDA	-0.13	0.24	0.26	0.77
GVBsrPBE, TDA*	-0.15	0.25	0.26	0.74

2.2 SSCCs: comparison to CC3 benchmark values

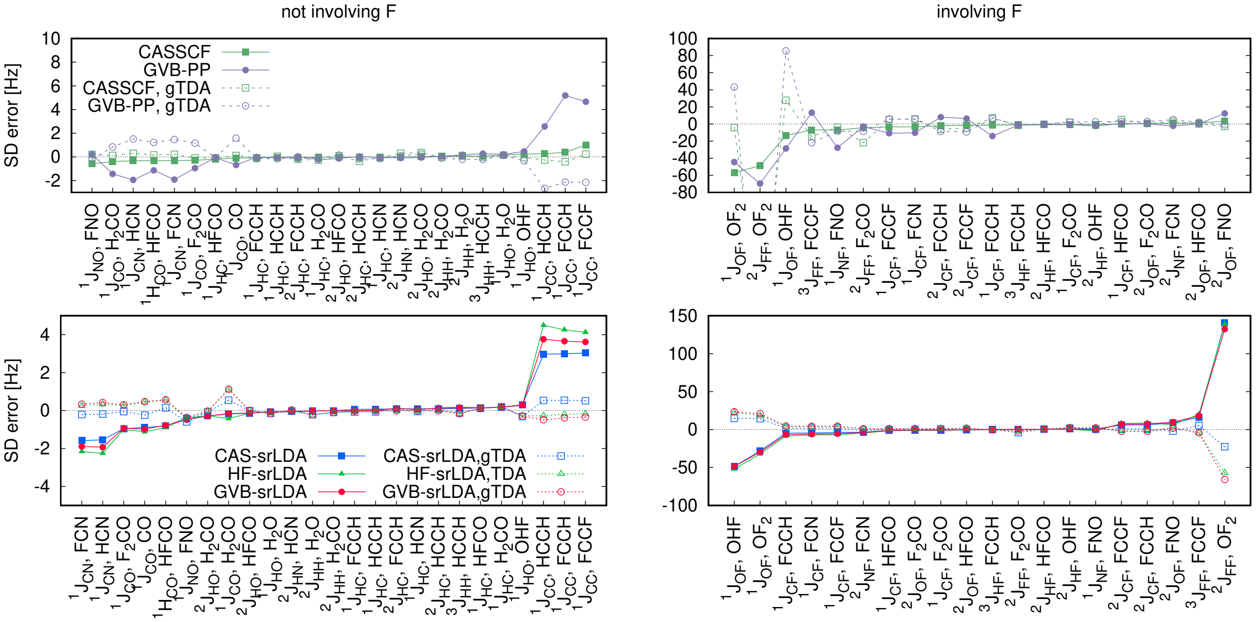

Figure S9: Errors relative to CC3 in the **SD contributions** to the SSCCs obtained in the "full E[2]" and gTDA approaches, sorted from the most negative to most positive error in CASSCF/CASsrLDA calculations. Lines are drawn to guide to eye.

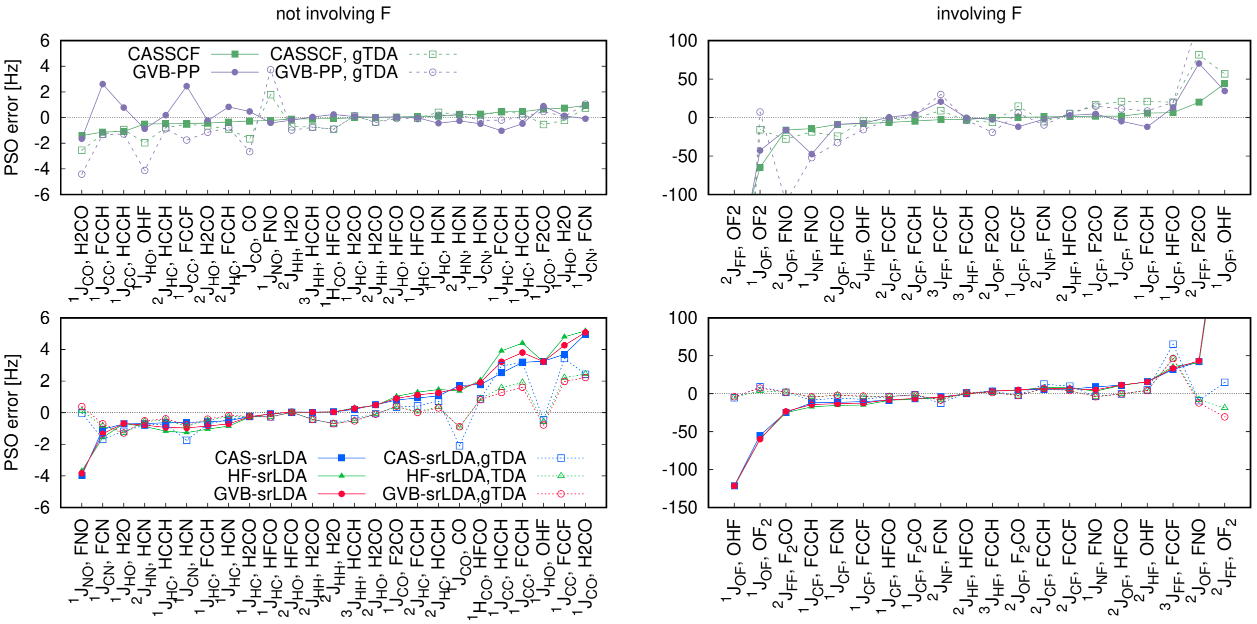

Figure S10: Errors relative to CC3 in the **PSO contributions** to the SSCCs obtained in the "full E[2]" and gTDA approaches, sorted from the most negative to most positive error in CASSCF/CASsrLDA calculations. Lines are drawn to guide to eye. All srDFT contributions calculated using $\mu = 0.4$ bohr^{-1}.

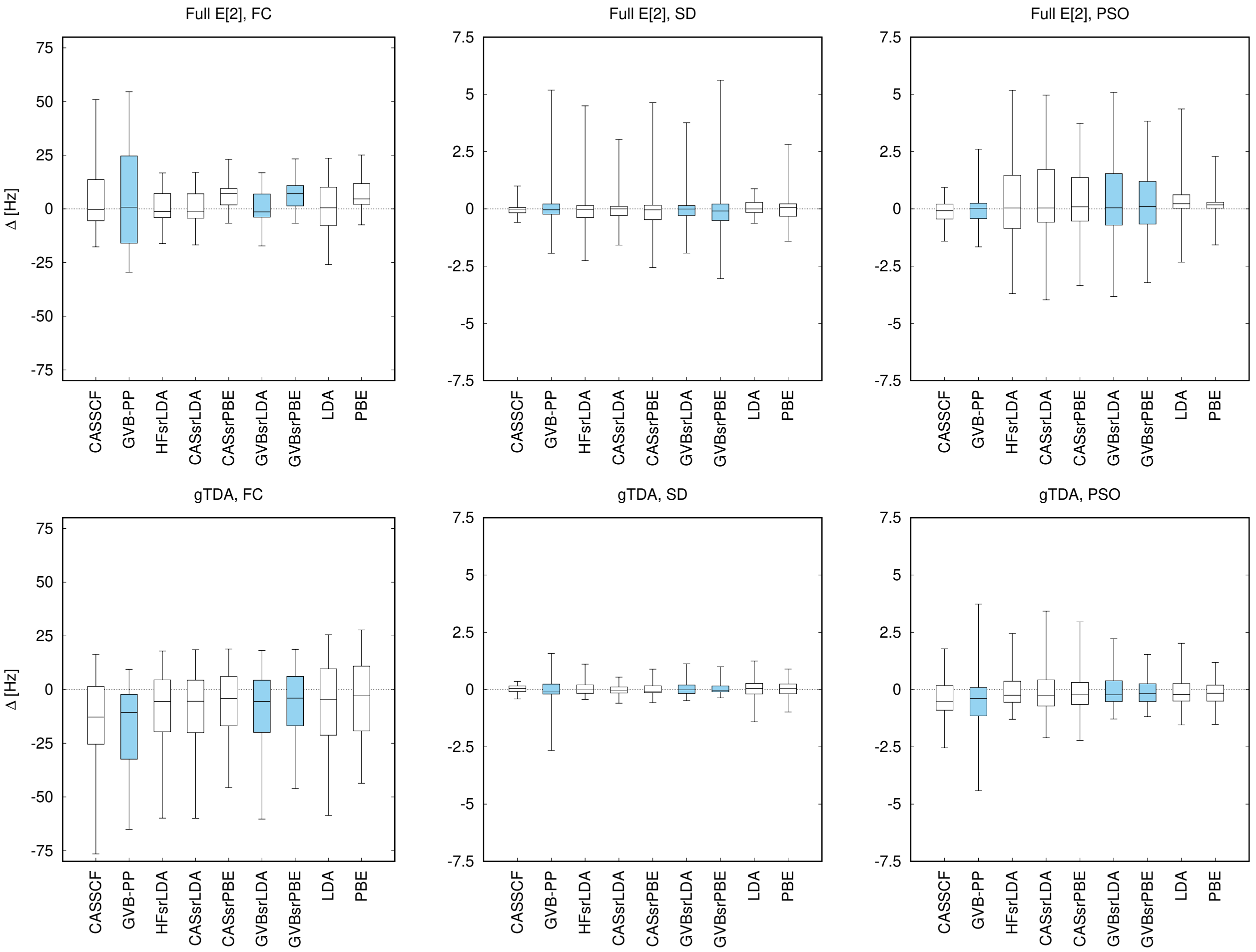

Figure S11: MAD in Hz for the FC, SD, and PSO contributions with respect to the CC3 reference for all couplings **not involving fluorine**. Note the different scales for the FC results compared to SD and PSO. The colored line denotes the median value, the box is the middle 25% to the 75% quantile, and the whiskers denote max and min. GVB-PP and GVB-srDFT results marked in blue.

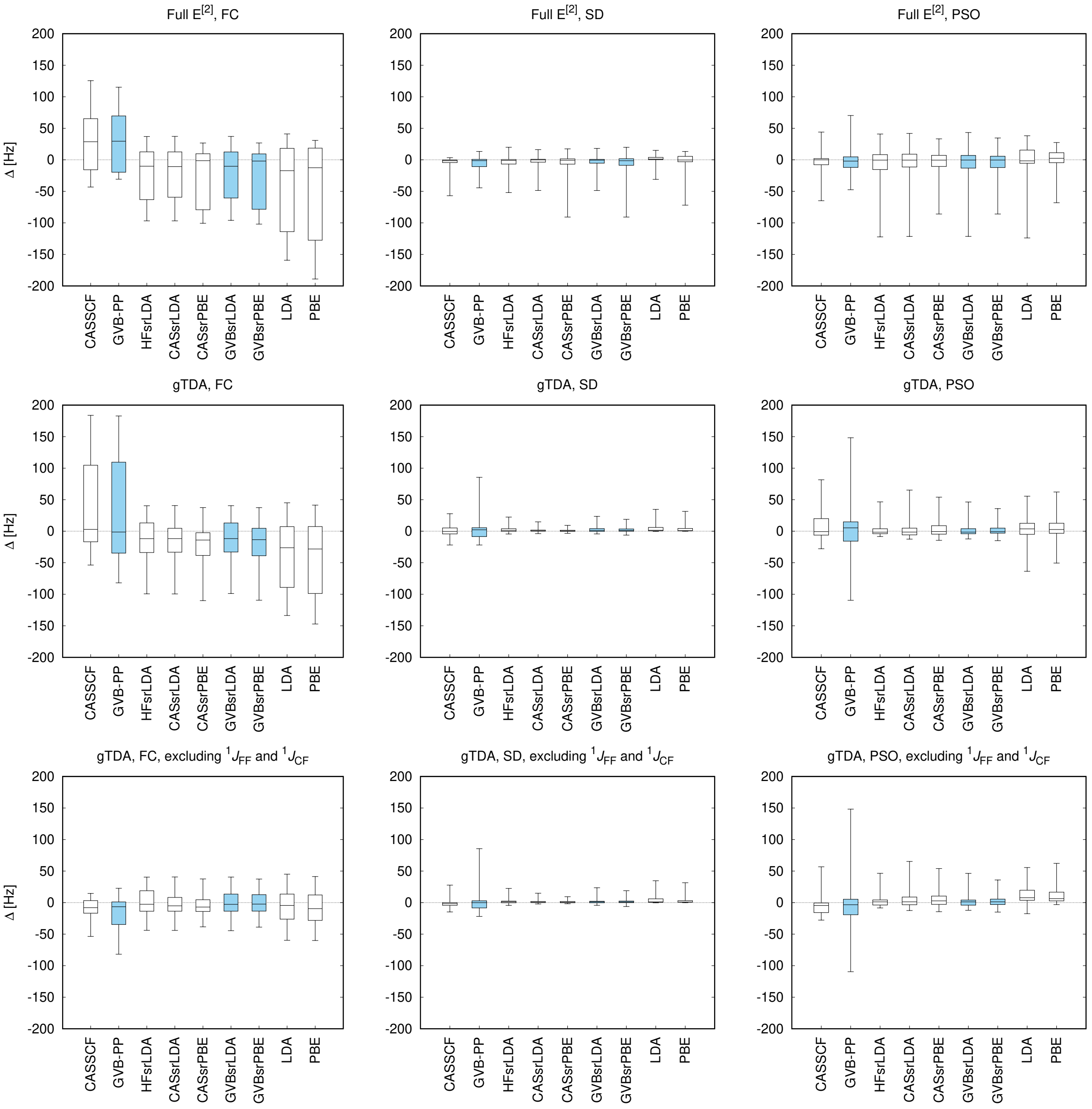

Figure S12: MAD in Hz for the FC, SD, and PSO contributions with respect to the CC3 reference for all couplings **involving fluorine**. The first and second rows excludes the $^2J_{\mathrm{FF}}$ coupling from OF_2 as an outlier due to the PSO term being more than 200 Hz different from the reference CC3 calculation. The colored line denotes the median value, the box is the middle 25% to the 75% quantile, and the whiskers denote max and min. GVB-PP and GVB-srDFT results marked in blue.

2.3 Transition metal complexes with F ligands, MF_6^{-z}

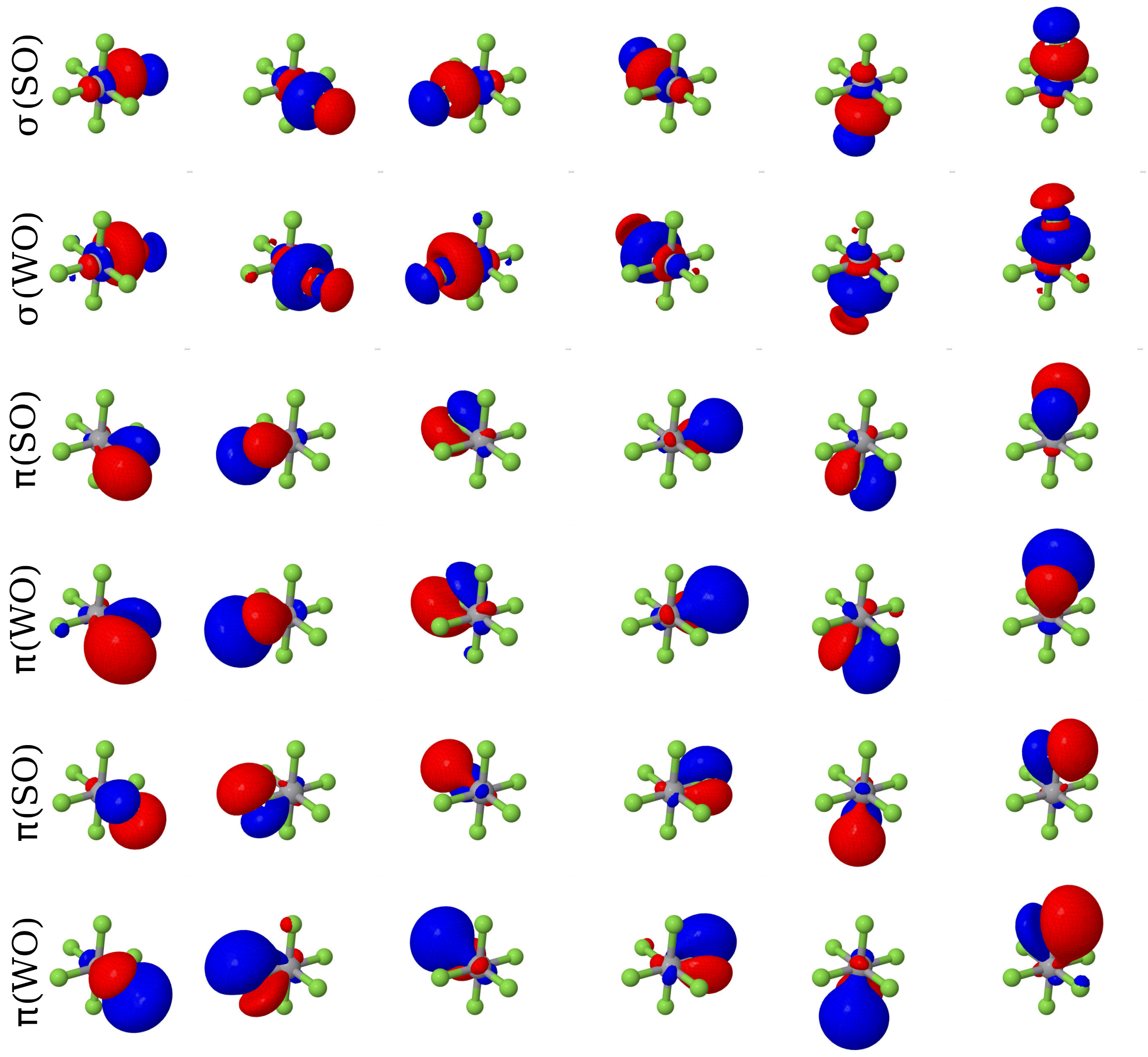

Figure S13: Visualization of VF_6^- geminals obtained in GVB-PP calculations. SO and WO labels refer to strongly and weakly occupied orbitals, respectively. Basis set is aug-cc-pVTZ.

Table S4: Calculated SSCCs for the VF_6^- complex. All srDFT calculations are with $\mu = 1.0$ bohr^{-}1. Energy unit is Hz.

Method	FC	PSO	SD	DSO	Isotropic
GVB-PP	409.4	−317.2	15.76	0.866	108.8
GVB-srLDA	370.3	−392.2	17.15	0.868	−3.867
GVB-srPBE	377.3	−378.6	17.70	0.868	17.19
CASSCF	441.0	−465.8	7.721	0.869	−16.19
CAS-srLDA	381.0	−537.7	7.019	0.868	−148.8

CAS-srPBE	387.7	−519.6	7.289	0.869	−123.8
HF-srLDA	379.7	−379.1	18.57	0.866	19.98
LDA	283.7	−355.5	22.46	0.870	−48.53
Benchmark					88.44

gTDA	FC	PSO	SD	DSO	Isotropic
GVB-PP	275.5	−235.2	12.00	0.866	53.17
GVB-srLDA	310.6	−301.5	14.54	0.868	24.50
GVB-srPBE	307.0	−294.3	14.53	0.868	28.05
CASSCF	271.3	−250.5	12.68	0.869	34.27
CAS-srLDA	318.2	−313.1	15.46	0.868	21.41
CAS-srPBE	313.9	−306.6	15.41	0.869	23.62
HF-srLDA	318.0	−298.1	15.13	0.866	35.85
LDA	261.6	−351.3	21.11	0.870	−67.73
Benchmark					88.44

singlet gTDA	FC	PSO	SD	DSO	Isotropic
GVB-PP	409.4	−235.2	15.76	0.866	190.9
GVB-srLDA	370.3	−301.5	17.15	0.868	86.81
GVB-srPBE	377.3	−294.3	17.70	0.868	101.5
CASSCF	441.0	−250.5	7.721	0.869	199.1
CAS-srLDA	381.0	−313.1	7.019	0.868	75.80
CAS-srPBE	387.7	−306.6	7.280	0.869	89.23
HF-srLDA	379.7	−298.1	18.57	0.866	101.0
HF-srPBE	386.4	−292.2	18.95	0.866	114.0
LDA	283.7	−351.3	22.46	0.870	−44.30
Benchmark					88.44

triplet gTDA	FC	PSO	SD	DSO	Isotropic
GVB-PP	275.5	−317.2	12.00	0.866	−28.85
GVB-srLDA	310.6	−392.2	14.54	0.868	−66.18
GVB-srPBE	307.0	−378.6	14.53	0.868	−56.27
CASSCF	271.3	−465.8	12.68	0.869	−181.0
CAS-srLDA	318.2	−537.7	15.46	0.868	−203.2
CAS-srPBE	313.9	−519.6	15.41	0.869	−189.4
HF-srLDA	318.0	−379.1	15.13	0.866	−45.16
HF-srPBE	313.8	−370.8	15.07	0.866	−41.06
LDA	261.6	−355.5	21.11	0.870	−71.96

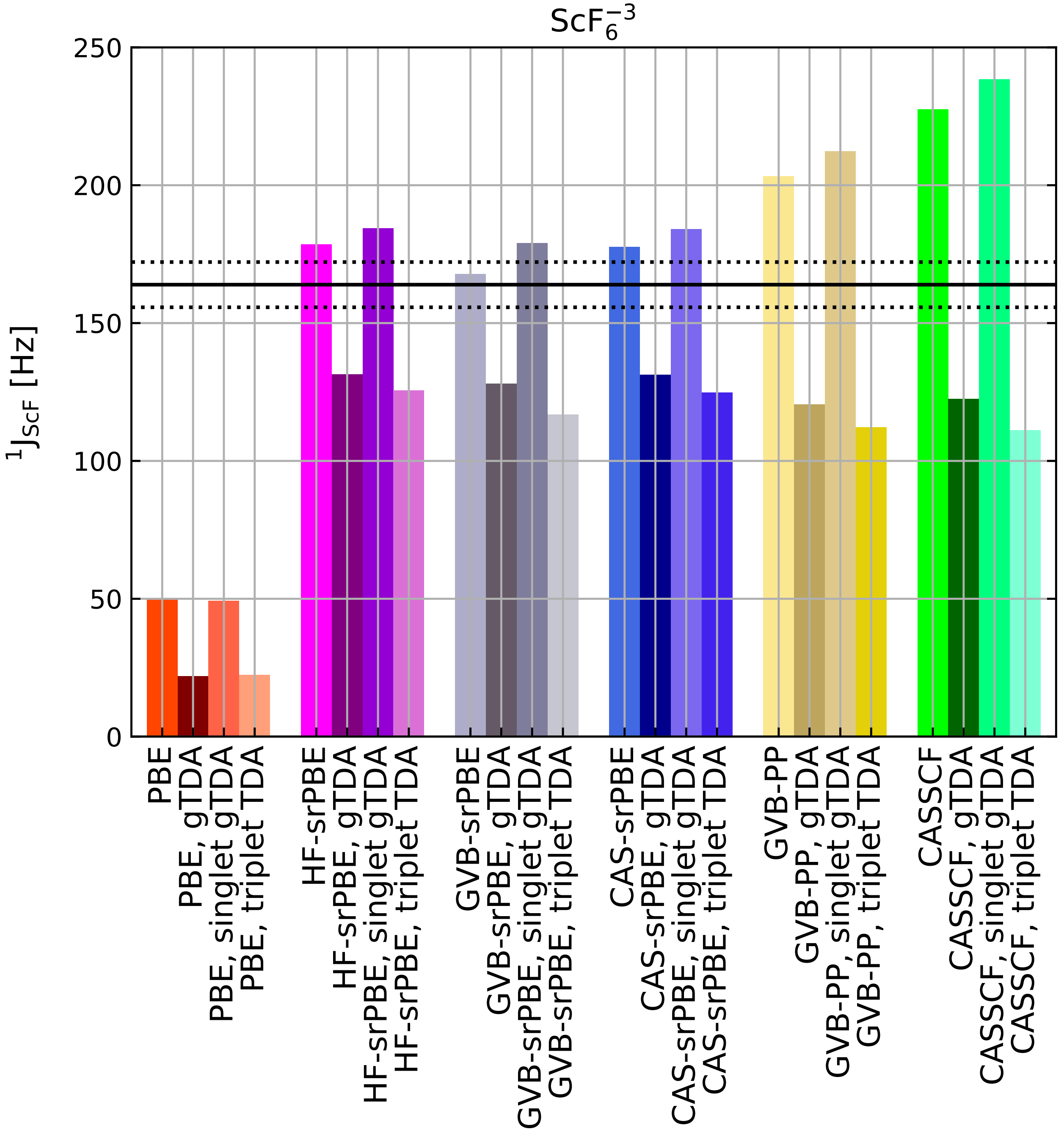

Figure S14: Calculated SSCCs for the ScF_6^{-3} complex. All the srDFT calculations are with $\mu = 1.0$ bohr. The black dashed lines represent the reference coupling ± 5 Hz. For each method, four results are reported: full $E[2]$, full gTDA, only gTDA in singlet response, and only gTDA in triplet response.

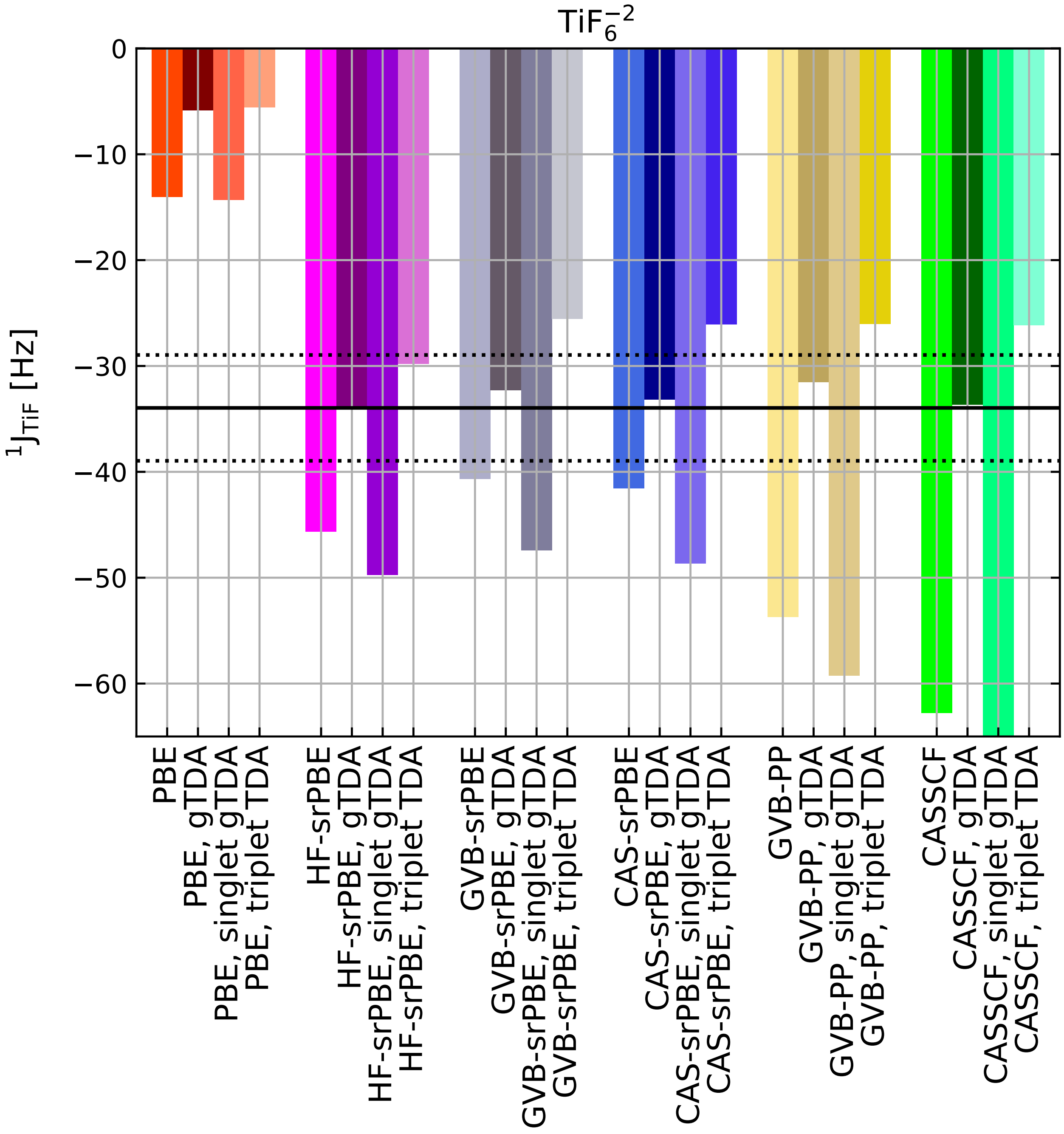

Figure S15: Calculated SSCCs for the TiF_6^{-2} complex. All the srDFT calculations are with $\mu = 1.0$ bohr. The black dashed lines represent the reference coupling ±5 Hz. For each method, four results are reported: full $E[2]$, full gTDA, only gTDA in singlet response, and only gTDA in triplet response.

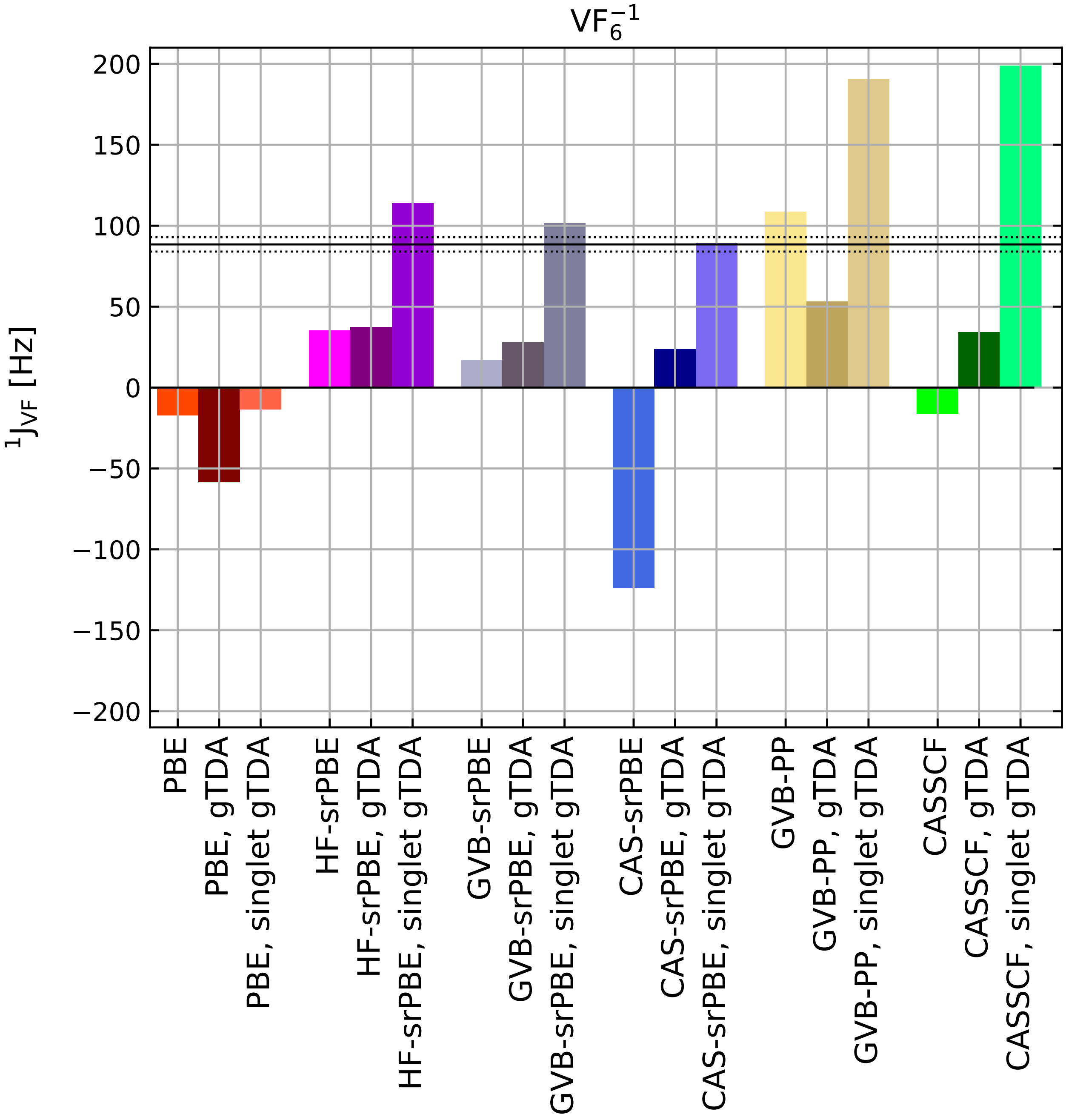

Figure S16: Calculated SSCCs for the VF_6^{-1} complex. All the srDFT calculations are with $\mu = 1.0$ bohr. The black dashed lines represent the reference coupling ± 5 Hz. For each method, three results are reported: full $E[2]$, full gTDA, and only gTDA in singlet response.
